# Brain Perihematoma Genomic Profile Following Spontaneous Human Intracerebral Hemorrhage

**DOI:** 10.1371/journal.pone.0016750

**Published:** 2011-02-02

**Authors:** Anna Rosell, Anna Vilalta, Teresa García-Berrocoso, Israel Fernández-Cadenas, Sophie Domingues-Montanari, Eloy Cuadrado, Pilar Delgado, Marc Ribó, Elena Martínez-Sáez, Arantxa Ortega-Aznar, Joan Montaner

**Affiliations:** 1 Neurovascular Research Laboratory and Department of Neurology, Universitat Autònoma de Barcelona, Institut de Recerca, Hospital Vall d'Hebron, Barcelona, Spain; 2 Stroke Unit and Department of Neurology, Universitat Autònoma de Barcelona, Hospital Vall d'Hebron, Barcelona, Spain; 3 Neuropathology Unit, Department of Pathology, Universitat Autònoma de Barcelona, Hospital Vall d'Hebron, Barcelona, Spain; Hospital Vall d'Hebron, Spain

## Abstract

**Background:**

Spontaneous intracerebral hemorrhage (ICH) represents about 15% of all strokes and is associated with high mortality rates. Our aim was to identify the gene expression changes and biological pathways altered in the brain following ICH.

**Methodology/Principal Findings:**

Twelve brain samples were obtained from four deceased patients who suffered an ICH including perihematomal tissue (PH) and the corresponding contralateral white (CW) and grey (CG) matter. Affymetrix GeneChip platform for analysis of over 47,000 transcripts was conducted. Microarray Analysis Suite 5.0 was used to process array images and the Ingenuity Pathway Analysis System was used to analyze biological mechanisms and functions of the genes. We identified 468 genes in the PH areas displaying a different expression pattern with a fold change between −3.74 and +5.16 when compared to the contralateral areas (291 overexpressed and 177 underexpressed). The top genes which appeared most significantly overexpressed in the PH areas codify for cytokines, chemokines, coagulation factors, cell growth and proliferation factors while the underexpressed codify for proteins involved in cell cycle or neurotrophins. Validation and replication studies at gene and protein level in brain samples confirmed microarray results.

**Conclusions:**

The genomic responses identified in this study provide valuable information about potential biomarkers and target molecules altered in the perihematomal regions.

## Introduction

Spontaneous intracerebral hemorrhage (ICH) is a sudden devastating form of stroke associated with poor neurological outcome and high mortality rates. ICH constitutes a major public health problem with an annual incidence of 10–30 cases per 100,000 population accounting for 2 million strokes worldwide each year [1]. However, nowadays no satisfactory treatment exists. Promising clinical trials have recently failed to demonstrate long-term benefits by preventing hematoma expansion using recombinant activated Factor VII (rFVIIa) [2], although a subset of younger patients without extensive bleeding at baseline can benefit from rFVIIa if given within 2.5 hours of symptoms onset [3]. Hence, nowadays excellent medical care given at stroke units is the only action with potent and direct impact on ICH morbidity and mortality until a specific therapy is found to treat these patients [4].

Brain injury after ICH occurs in two phases: a sudden and initial mass effect of intraparenchymal blood which produces mechanical disruption of the neurons and glial cells and a secondary brain injury in surrounding tissue where hematoma expansion, edema, blood brain barrier disruption, apoptosis and inflammation can occur. Neuroimaging studies have demonstrated that hematomas are dynamic and might expand over time associated with cerebral edema and secondary brain injury. In this sense it is known that, on average, perihematomal edema volume increases acutely by approximately 75% during the first 24 hours after ICH [5] and chronically lasts up to day 14 [6]. Thus, to describe genomic changes in the perihematomal areas could be an interesting approach to identify pathological processes that occur after ICH and contribute to perihematomal edema and injury expansion.

Two interesting studies have recently approached the genomic regulation after ICH by microarray studies [7], [8]. First Lu and colleagues investigated the brain genomics in a rat model of intracerebral hemorrhage induced by intrastriatal autologous blood infusion. Their study focused on the expression patterns between ICH and non-ICH rats identifying a large number of genes being regulated in the striatum and the cortical tissue. A major number of up-regulated genes were directly related to edema and cellular apoptosis. More recently Carmichael and colleagues studied by microarray technology the genomic patterns in acute ICH (<24 hours) compared to healthy brains. In this study ICH tissue was obtained when adherent tissue to the clot was incidentally removed during hematoma evacuation in patients included in the Hemorrhage Evacuation using MR-guided Endoscopy Surgery—HEME Surgery trial and control tissue was obtained from autopsy cases that died from cardiopulmonary arrest. The authors report an upregulation of pro-/anti-inflammatoy networks and downregulation of neuronal signaling pathways.

Our aim was to identify the molecular profiles that contribute to genomic expression patterns after human spontaneous ICH and to compare our results with other human studies with similar approaches. The study has been designed to identify common expression patterns since patients' samples from several hours/days of stroke onset have been studied. Moreover, comparisons have been done in samples coming from the same individuals including differences between perihematomal and contralateral healthy tissues. Finally, a large number of genes has been validated and also replicated in other ICH cases to confirm consistency and relevance of our data. We believe that our findings present potential therapeutic targets and biomarkers for ICH and deserve further investigation.

## Methods

### Ethics statement

This study was approved by the Ethics Committee of the Vall d'Hebron Hospital and informed and written consent was acquired from all patients or relatives and from controls.

### Patients and samples

Brain samples from 4 deceased patients who had a supratentorial intracerebral hemorrhage within the previous 4 days were included in the microarray study. This study group included 2 women and 2 men with a median age of 79 (68–92) years. On autopsy and during macroscopic exam, perihematomal areas suspected to present edema were identified by an experienced neuropathologist using the more recent neuroradiological images available. Samples from perihematomal areas (PH), contralateral grey matter (CG) and contralateral white matter (CW) were obtained within the first 5 hours after death, snap frozen in liquid nitrogen and stored at −80°C until RNA isolation. For the replication study four additional patients who had also a supratentorial intracerebral hemorrhage in the previous 2 days were selected including 4 men with a median age of 79 (69–90) years and samples were obtained within the first 18 hours after death and stored as previously described. Demographic and tissue sampling data are listed in Table 1.

**Table 1:** Demographic and tissue sampling data.

Case	Sex	Age	Time S-E	Time E-T	Study
27	F	72	12 h	5 h	Microarray, Validation and tissue homogenate
28	F	95	39 h	2,5 h	Microarray, Validation and tissue homogenate
30	M	67	17 h	3 h	Microarray, Validation and tissue homogenate
31	M	86	4 days	4 h	Microarray, Validation and tissue homogenate
10	M	92	15 h	5 h	Replication and tissue homogenate
11	M	68	18 h	4,5 h	Replication and tissue homogenate
17	M	74	2 days	5 h	Replication and tissue homogenate
20	M	85	5 h	18 h	Replication and tissue homogenate

F, female; M, male; S-E, Stroke to Exitus; E-T, Exitus to Tissue sampling.

Additionally, blood samples from 24 hemorrhagic strokes and 20 stroke-free controls at the time of blood sampling were collected in EDTA tubes and plasma was stored at −80°C for protein determination. All patients had an acute hypertensive intracerebral hemorrhage (ICH) and attended the emergency department of a teaching hospital. On admission, all patients underwent a cranial computed tomography (CT) scan and the National Institute of Health Stroke Scale (NIHSS) score was recorded to assess neurological status. Blood samples were obtained within the first 24 hours of stroke symptoms onset and a detailed history of vascular risk factors was obtained from each patient and control. Demographic characteristics and risk factors profile are described in Table 2.

**Table 2:** Demographic features of stroke patients and healthy volunteers.

	Healthy controls(n = 20)	Stroke patients(n = 24)	P value
**Age (years)**	69±12	69.2±12.7	0.995
**Male gender (%)**	36.8	62.5	0.095
**Smokers (%)**	21.1	16.7	0.714
**Hypertension (%)**	47.4	70.8	0.118
**Alcohol (%)**	0	16.7	0.069
**Diabetes (%)**	21.1	4.2	0.074
**Dyslipidemia (%)**	10.5	12.5	0.841
**Coronary artery disease (%)**	15.8	8.4	0.193
**Atrial fibrillation (%)**	0	20.8	0.039

### RNA purification and hybridization

RNA was isolated from frozen brain samples using the RNeasy® kit (Qiagen, Austin, TX, USA). Briefly, approximately 20–40 mg of frozen tissue was cut from the original piece and purification was conducted following manufacturers' instructions. RNaseZAP was used to clean all surfaces and material. The concentration and integrity of the total RNA was determined using the Bioanalyzer 2100 platform according to the manufacturers' protocols (Agilent, UK). Only RNA with a RIN (RNA integrity number) ≥5 was used for the RNA hybridization. An additional cycle of cDNA synthesizer and amplification was performed to all samples. Briefly, double-stranded complementary DNA (cDNA) was synthesized from total purified RNA and biotin-labeled complementary cRNA was synthesized from cDNA. Finally cRNA was hybridized to the Affymetrix GeneChip platform for analysis of over 47,000 transcripts (human U133 Plus 2.0). This was conducted by the genomics platform core facility of our institution. All data presented in this study is MIAME compliant and the raw data have been deposited in a MIAME compliant database, the NCBI's Gene Expression Omnibus (GEO), accessible through GEO Series accession number GSE24265 [9].

### Microarray Analysis

The goal of the study was to compare the gene expression patterns between perihematomal and contralateral healthy tissue from the same individuals. Then, to rule out the possibility of detecting inherent differences between white and grey matter not influenced by the disease, an indirect comparison was performed by independently comparing both white and grey matter from the contralateral hemisphere against the perihematomal tissue and then comparing white and grey matter between them. Therefore, 3 comparisons of gene expression were calculated per individual: PH vs. CG, PH vs. CW and CW vs. CG.

The images were processed with the Microarray Analysis Suite 5.0 (Affymetrix, USA). All samples demonstrated characteristics of high-quality cRNA (3′/5′ ratio of probe sets for glyceraldehyde-3-phosphate dehydrogenase and beta-actin of <1.5) and were subjected to subsequent analysis. Raw expression values obtained directly from. CEL files were preprocessed using the RMA (Robust Multi-Array) method, a three-step process which integrates background correction, normalization and summarization of probe values. These normalized values were the basis for all the analyses. The selection of differentially expressed genes between conditions was based on a linear model analysis with empirical Bayes moderation of the variance estimates. In order to deal with the multiple testing issues derived from the fact that many tests (one per gene) are performed simultaneously, p-values were adjusted to obtain strong control over the false discovery rate (FDR) using the Benjamini and Hochberg method. Array quality was measured by a series of parameters which are usually provided by the core facility to confirm that the arrays had been properly hybridized. To check data quality standard approaches for quality control were conducted (standard quality controls based on Affymetrix original methods and Probe-level models) plus background correction, normalization and filtering. All the statistical analyses, were done using the free statistical language R and the libraries developed for microarray data analysis by the Bioconductor Project (www.bioconductor.org).

A further analysis to identify the biological mechanisms, pathways and functions and the most relevant genes was performed using the Ingenuity Pathways Analysis (IPA) software and database (www.ingenuity.com). Briefly, the dataset containing gene identifiers and corresponding fold changes was uploaded into the web-delivered application and each gene identifier was mapped to its corresponding gene object. The threshold for a significant association was determined by the –log (0.05), becoming significant any score above 1.3.

### Quantitative Real-Time Polymerase Chain Reaction (qRT-PCR)

A total of 8 patients were studied for gene validation (n = 4) and replication (n = 4) by qRT-PCR technique (see table 1). Twenty genes were selected including those that were most up-regulated (*IL-8*, *CCL20*, *CXCL5*, *C15ORF48*, *SERPINE-1*, *TFPI2*, *CD163*, *CXCL1*, *TMEM49*) or down-regulated (*LHX2*, *MUM1L1*, *PRDM16*, *NTRK2*, *PPARGC1A*, *ERBB4*, *CRB1*, *LRRC8B* and *KCNAB1*) in PH areas and displaying a highlighted role in the functional pathways analysis. *BCAT-1*, differentially expressed presenting the lowest Adj.P.value and *GABBR2*, identified as a neuronal marker of grey matter and found up-regulated in our CG areas, were also included.

Briefly, cDNA synthesis was performed using High-Capacity cDNA Reverse Transcription Kit (Applied Biosystems Inc, USA). mRNA levels were determined by RT-PCR using a standard TaqMan® PCR kit protocol and TaqMan fluorogenic probes with a 7500 Real Time PCR System (Applied Biosystems). The probe location of each particular gene is stated in table 3 and the Cyclophilin A (*PPIA*) gene (Hs99999904_m1) was used as housekeeping gene to normalize the results. All reactions were run in triplicate on 96-well plates, using a unique sample as endogenous calibrator control in each one, and analyzed using the Applied Biosystems SDS 7500 system software (Applied Biosystems). The RQ values were calculated by use of the Livak equation: RQ  = 2−ΔΔCt and the results are expressed as a ratio depending on the calibrator sample used in all experiments.

**Table 3:** List of selected genes and primers for quantitative RT-PCR.

Gene name	LogFC	Adj.P.value	Primer	Studied areas
***BCAT-1***, branched chain amino-acid transaminase 1	3.45	0.0006	Human *BCAT-1* Hs00194075_m1	PH vs. CG and CW
***C15ORF48***, chromosome 15 open reading frame 48	4.38	0.0046	Human *C15ORF48* Hs00260902_m1	PH vs. CG and CW
***CCL20***, chemokine (C-C motif) ligand 20	4.81	0.0051	Human *CCL20* Hs00355476_m1	PH vs. CG
***CD163***, CD163 molecule	4.16	0.0379	Human *CD163* Hs01016661_m1	PH vs. CW
***CRB***, crumbs homolog 1	−2.31	0.0039	Human *CRB1* Hs00201372_m1	PH vs. CG
***CXCL1***, chemokine (C-X-C motif) ligand 1	4.01	0.0037	Human *CXCL1* Hs00236937	PH vs. CG and CW
***CXCL5***, chemokine (C-X-C motif) ligand 5	4.45	0.0346	Human *CXCL5* Hs00171085_m1	PH vs. CG and CW
***ERBB4***, v-erb-a erythroblastic leukemia viral oncogene homolog 4	−2.33	0.0098	Human *ERBB4* Hs00171783_m1	PH vs. CG
***GABBR2***, gamma-aminobutyric acid (GABA) B receptor, 2	−3.49	0.0028	Human *GABBR2* Hs01554998_m1	CW vs. CG
*IL-8*, Interleukin-8	5.16	0.0084	Human *IL-8* Hs99999034_m1	PH vs. CG
***KCNAB1***, potassium voltage-gated channel, beta member 1	−2.20	0.0142	Human *KCNAB1* Hs00963155_m1	PH vs. CG
***LHX2***, LIM homeobox 2	−3.74	0.0079	Human *LHX2* Hs00180351_m1	PH vs. CG
***LRRC8B***, leucine rich repeat containing 8 family, member B	−2.27	0.0027	Human *LRRC8B* Hs00372164_m1	PH vs. CG
***MUM1L1***, melanoma associated antigen (mutated) 1-like 1	−2.82	0.0014	Human *MUM1L1* Hs00327838_m1	PH vs. CG
***NTRK2***, neurotrophic tyrosine kinase, receptor, type 2	−2.54	0.0049	Human *NTRK2* Hs01093103_m1	PH vs. CG
***PPARGC1A***, peroxisome proliferator-activated receptorgamma, coactivator 1 alpha	−2.38	0.0248	Human*PPARGC1A* Hs01016724_m1	PH vs. CG
***PRDM16***, PR domain containing 16	−2.72	0.0134	Human *PRDM16* Hs00922674_m1	PH vs. CG
***SERPINE1***, serpin peptidase inhibitor, clade E (nexin,plasminogen activator inhibitor type 1), member 1	4.32	0.0034	Human *SERPINE1* Hs00167155_m1	PH vs. CG and CW
***TFPI2***, tissue factor pathway inhibitor 2	4.23	0.0484	Human *TFPI2* Hs00197918_m1	PH vs. CG and CW
***TMEM49***, transmembrane protein 49	3.91	0.0072	Human *TMEM49* Hs00229548_m1	PH vs. CG and CW

PH (perihematoma), CW (contralateral white) and CG (contralateral grey).

Statistical analysis was conducted using the Statistical Products and Service Solution (SPSS) package 15.0. Statistical significance for intergroup differences was assessed by the Mann–Whitney U and Kruskal–Wallis tests. A p-value lower than 0.05 was considered statistically significant. Median values and interquartile range are displayed in box-plot graphs.

### IL-8 Enzyme Linked InmunoSorbent Assay

Validation and replication studies were also performed at the protein level for IL-8. For this purpose the Enzyme Linked Inmunosorbent Assay (ELISA) kit from Invitrogen (Human IL-8 Ultrasensitive; Camarillo, CA) was used for the *in vitro* quantitative determination of IL-8 in human brain tissue (n = 8) and plasma (n = 20 controls and n = 24 ICH) according to manufacturer's instructions. Tissue homogenates were obtained as previously described [10] from the same ICH patients included in the replication and validation studies that table 1 indicates. Each sample was analyzed twice and the mean of the two values was used. The mean intra-assay coefficient of variation of optical densities was lower than 30% for all samples measured. For tissue samples total protein content of brain homogenates was determined by the BCA method and equal amounts (25 micrograms) were assayed and results are expressed as pg/mL/µg protein. For plasma samples results are expressed as pg/mL.

Statistical analysis was conducted using the SPSS package 15.0. Statistical significance for intergroup differences was assessed by the Student t, Mann–Whitney U, and Kruskal–Wallis tests. A p-value lower than 0.05 was considered statistically significant. Median values and interquartile range are displayed in graphs.

## Results

### Preprocessing and data exploration

One array from the contralateral white matter presented an unusual performance. After detailed exploration it was removed before the final analysis was performed (sample 30CW). The remaining data was properly analyzed using standard techniques. Briefly, quality control parameters and plots shown in figure S1 indicate that the quality of the arrays was good, including acceptable and homogenous RNA degradation and signal distribution and a relatively low background (figure S1A–C). Once the quality of the data was verified, they were normalized in order to eliminate systematic biases. Homogeneity of the samples was analyzed to check if similarities existed between samples of the same areas (figure S1D). A plot of the first two principal components from a principal component analysis (PCA) was used to show the overall structure of the data and we could see how replicate samples tend to group together, indicating general similarity in overall expression patterns (figure S1E). At the same time, hierarchical clustering with Euclidean distance was used to form the groups based on raw data showing that there was certain homogeneity since samples from the same areas were grouped into close clusters (figure S1F).

### Transcripts showing differential expression profile

**Figure 1:**
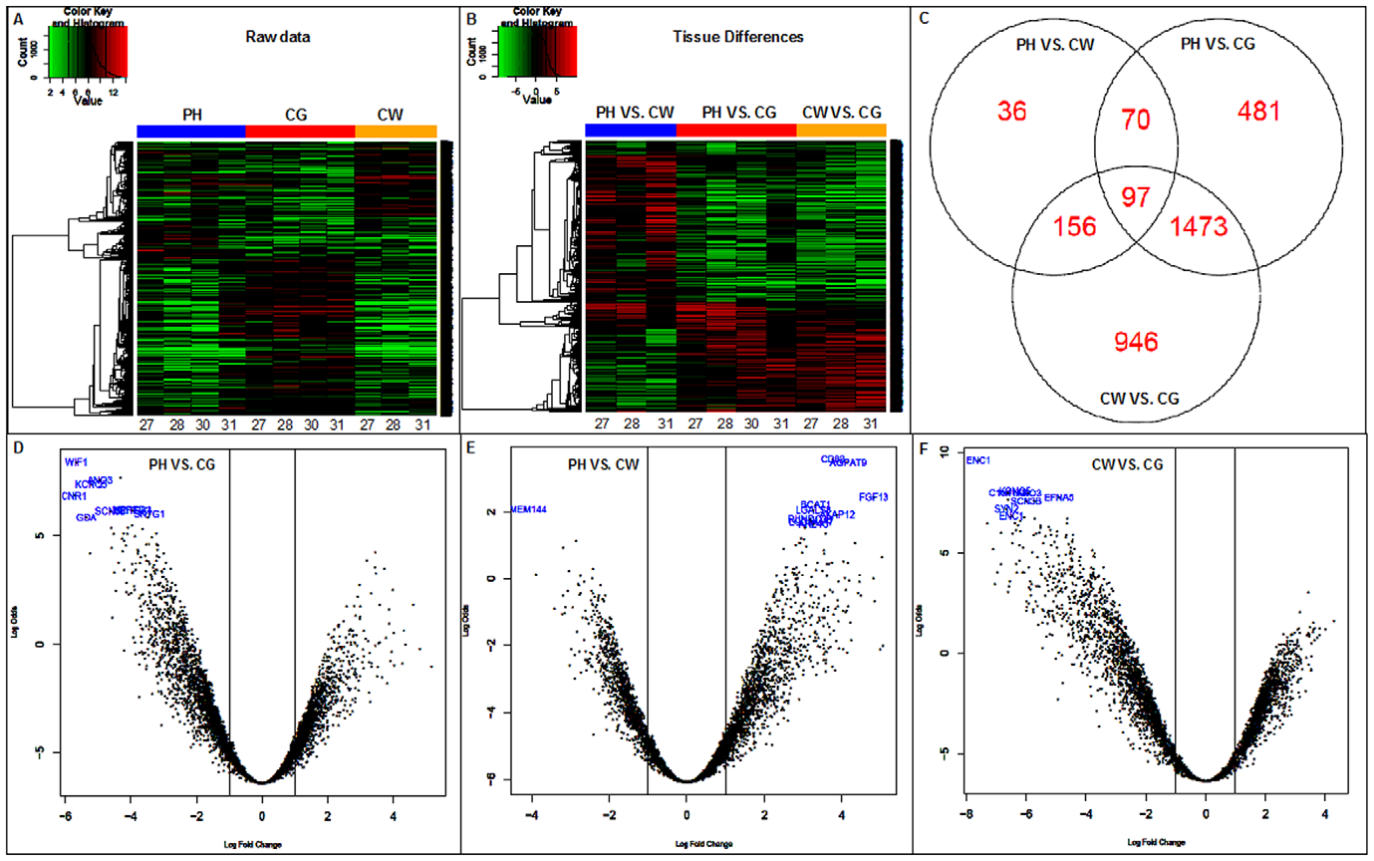
Raw data from the microarray analysis. **A** and **B**) heat maps illustrating gene expression, **C**) Venn diagram of a total of 3,259 genes significantly regulated; **D**, **E** and **F**) volcano plots arranging the genes significantly expressed corresponding to the three comparison areas along dimensions of biological and statistical significance. PH (perihematoma), CW (contralateral white) and CG (contralateral grey).

In order to increase statistical power and reduce unnecessary noise not all genes were analyzed but only those which were considered to be expressed and showed some variability. This left a total of 4,136 genes, and of these 3,259 presented significant differences adjusted by the false discovery rate. Heat maps (color-coded graphs with samples in columns and genes in rows) were used to visualize them. Figure 1 illustrates the heat map of individual gene expression (figure 1A) and of differences between paired areas (figure 1B) showing that there are groups of co-regulated genes with a homogeneous expression pattern between different individuals.

The analysis showed that 359 genes were differentially expressed in the PH vs. CW group, 2,121 between PH vs. CG and 2,672 between CW vs. CG areas (adj.P.Val <0.05 for all groups), see figure 1C. Significant genes were also graphically highlighted using the volcano plots which arrange genes along dimensions of biological and statistical significance (the horizontal dimension is the fold change between the two groups and the vertical axis represents the p-value). Then the first axis indicates biological impact of the change and the second indicates the statistical evidence of the change, see figure 1D–F.

Importantly, for further analysis all genes showing significant differences in their expression between CW vs. CG groups were ruled out since they most likely represent inherent differences in healthy tissue due to different cellular populations and physiological functions (total of 2,672). This indirect comparison allowed us to discard potential mistakes and indicated that our perihematomal areas were mostly composed by white matter. Indeed, our results demonstrate that several transcripts reported to be good neuronal and glial markers of grey and white matter in human brain samples [11] were found differentially expressed between CW and CG (see table S1), being potential confounders if not eliminated from the final list of genes.

Finally, we identified 468 genes in the PH areas displaying a different expression pattern with a fold change between −3.74 and +5.16 when compared to the contralateral areas (291 were overexpressed and 177 were underexpressed). Of them, 6.5% of the genes represented differences between PH vs. CW, 82.5% between PH vs. CG and 11% between PH and both control areas. The complete list of genes is published as table S2 with its corresponding logFold Change and Adj.P.Value.

### Pathway and network analysis

Ingenuity Pathways Analysis is a knowledge repository of networks and biological relationships that has been manually created from over 200,000 full texts studying approximately 10,000 human genes. For genes with multiple probe sets the one with the highest logFold Change value was chosen to represent the gene (differences of expression between different probes were not significant, data not shown). A total of 468 probe sets representing the total number of differentially expressed identified genes were introduced into the IPA system to model and analyze the ontology pathways: of these 440 were mapped in the system, 134 were network eligible molecules and 303 were identified in a pathway.

**Figure 2:**
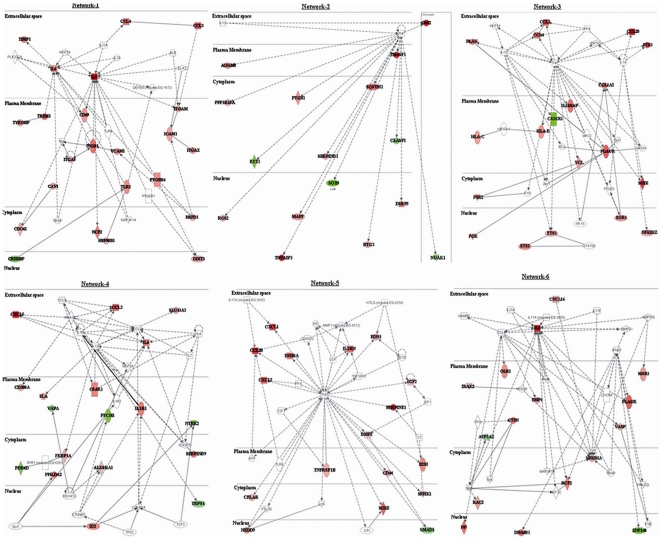
Most relevant molecular networks of interacting genes. The intensity of genes (node) color in the networks indicates the degree of downregulation (green) or upregulation (red) of gene expression; continuous lines indicate a direct interaction and dashed line an indirect interaction.

The functional analyses generated through the use of IPA identified relationships between genes differentially expressed after ICH in the perihematomal areas in a total of 19 networks. Eight of these networks were represented with more than 15 molecules involved with functions related to cell-to-cell signaling and interaction, antigen presentation, cell-mediated immune response, immune cell trafficking, cellular growth and proliferation or humoral immune response. Most relevant network relationships between molecules are represented in figure 2.

The gene expression pattern led a total of 137 canonical pathways, 48 of them showing a significant association as seen in table S3. The most relevant ones were related to cytokines (IL-6, IL-8, IL-10, IL-17) but also to leukocyte extravasation, TREM-1, integrin, TGF, p38MAPK or axonal guidance signaling. Within the large list of genes identified in our study, the IPA analysis highlighted several genes for being highly up or downregulated and for being central molecules in the created networks. These were *IL-8*, *CCL20*, *CXCL5*, *C15ORF48*, *SERPINE1*, *TFPI2*, *CD163*, *CXCL1* and *TMEM49* for up-regulated genes in the perihematomal areas and *FOXG1*, *LHX2*, *MUM1L1*, *PRDM16*, *NTRK2*, *PPARGC1A*, *ERBB4*, *CRB1*, *LRRC8B* and *KCNAB1* for downregulated genes in the perihematomal areas. Biofunctions related to these genes and networks ranked by significance correspond to cellular growth and proliferation [-log (pvalue) = 18.35], cell death [-log (pvalue) = 17.17], cellular development [-log (pvalue) = 13.82], cell-to-cell signaling and interaction [-log (pvalue) = 13.67], cellular movement [-log (pvalue) = 12.97], tissue development [-log (pvalue) = 12.89], cell morphology [-log (pvalue) = 12.22] or cell-mediated immune response [-log (pvalue) = 9.84] among others.

### Validation and replication of microarray data

**Figure 3:**
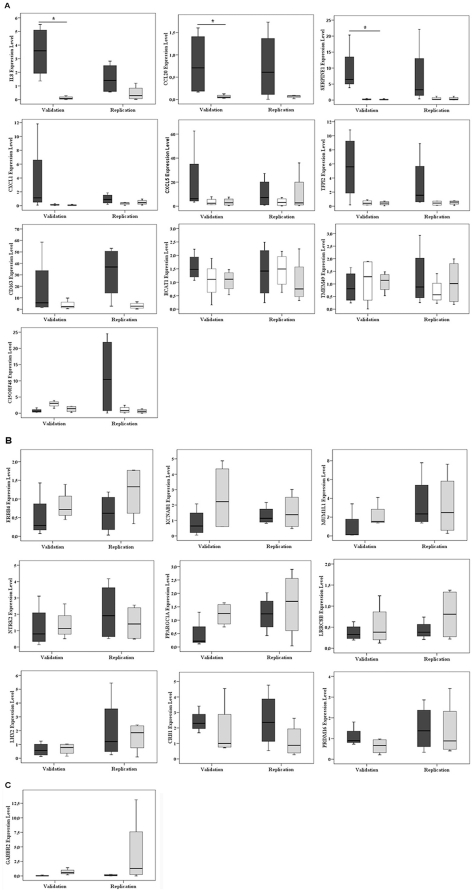
Complete quantitative RT-PCR validation and replication studies of overexpressed genes (A), underexpressed genes (B) and control-gene of grey matter (C). Validation study was statistically significant for *IL-8*, *CCL20* and *SERPINE-1*. Black bars represent PH (perihematoma), white bars CW (contralateral white) and grey bars CG (contralateral grey). Only the comparisons found differentially expressed in the main microarray study (as indicated in table 3) are shown here. Expression level is relative to calibrator sample *p<0.05.

Our results validate the overexpression patterns identified for *IL-8*, *CCL20* and *SERPINE-1* since gene expression was also elevated in perihematomal areas by qRT-PCR (p<0.05, see figure 3A). Although the replication study in a new group of samples did not show significant differences of these genes they were markedly increased in the perihematomal areas compared to the corresponding contralateral (see figure 3A). Likewise, the same overexpression pattern was confirmed for *CXCL1*, *CXCL5*, *TFPI2*, *CD163* in both validation and replication studies but not for *BCAT-1*, *TMEM49* and *C15ORF48*.

Regarding underexpressed genes most of them showed the same pattern found in the microarray results (*ERRB4*, *KCNAB1*, *PPARGC1A*, *LHX2* and *LRRC8B*) as seen in figure 3B. Only the validation but not the replication study was confirmed for *MUM1L1* and *NTRK2*. On the other hand, *CRB1* and *PRDM16* underexpression patterns in the perihematomal areas could not be confirmed by qRT-PCR as seen in figure 3B.

Finally, the internal control-gene to differentiate between white and grey matter (*GABBR2*) confirmed higher expression levels in grey matter than in white matter (figure 3C).

### Validation at the protein level

**Figure 4:**
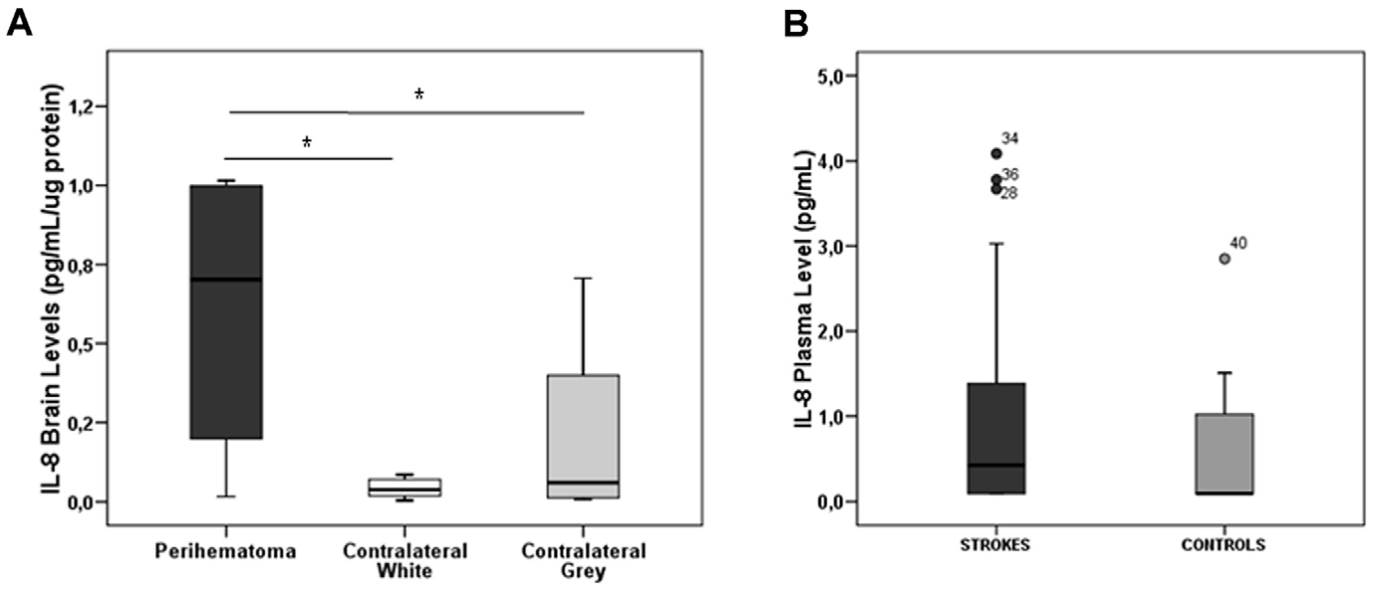
Validation studies at the protein level. Box-plots show how increased levels of IL-8 were found in perihematoma areas compared to both contralateral white and grey areas (**A**) and also in plasma of ICH stroke patients compared to controls (**B**). *p<0.05.

IL-8 was selected as a candidate gene to be validated at the protein level for its highlighted role in several functional pathways and the confirmed validation by qRT-PCR as an overexpressed gene in the perihematomal area after ICH. Our results confirm that IL-8 was also overexpressed at the protein level in PH [0.7 (0.16–1.00) pg/mL/µg protein] compared to both CW [0.03 (0.01–0.07) pg/mL/µg protein] and CG [0.06 (0.01–0.43) pg/mL/µg protein] in tissue homogenates (p = 0.036 and p = 0.046, respectively), as figure 4A shows. Likewise, IL-8 level was found elevated in blood samples of acute hemorrhagic stroke patients compared to healthy subjects [0.43 (0.13–2.61) vs. 0.09 (0.09–1.15) pg/mL], though not significantly (p = 0.104, figure 4B). Demographic data and risk factors for patients and controls listed in Table 2 shows that only atrial fibrillation was significantly more present in stroke patients than in controls.

## Discussion

Our study reports the genes and pathways that are altered after ICH in perihematomal areas compared to contralateral healthy tissue in human samples. Overall, our data shows that more than 400 genes display different expression patterns, most of them being overexpressed and identified in networks and pathways related to inflammation and immune response (such as cell-to-cell signaling, antigen presentation or cell trafficking) but also related to cellular growth, proliferation or axonal guidance signaling. These results suggest potential targets to both decrease hematoma expansion and to potentiate neuroreparative processes beginning in the early stages of this devastating disease. Finally, some of our findings have been validated and replicated in other patients at gene and protein level suggesting also their potential role as ICH biomarkers.

Nowadays microarray studies offer a robust method for the analysis of whole genome mRNA expression patterns. These techniques might become a revolution in our understanding of the molecular mechanisms underlying physiological and pathological processes and a tool for identifying new targets for treatment. Several experimental studies have examined the alteration of gene expression in the postischemic rat brain using microarray technology [12], [13], while in humans both blood and brain genomic profiling have been investigated after ischemic stroke [14]–[17]. Another interesting study from Mitsios and colleagues investigated the dynamic changes in gene expression in brain samples following stroke and compared them with those obtained at similar time points following middle cerebral artery occlusion in rats [17]. In this study the authors report significant differences in gene expression profile between human stroke and the animal model pointing out that an experimental model could not completely reflect the pathophysiological process of stroke in humans.

Regarding ICH, a disease with no effective treatment at the present time, two investigations have used the microarray technology to study gene expression. Firstly, Lu and colleagues induced ICH by intrastriatal blood infusion in a rat model investigating expression patterns compared with saline-injected animals and more recently Carmichael and colleagues described the gene pattern profile of human perihematomal tissue of acute ICH compared with brain tissue of autopsy cases dying from cardiopulmonary arrest [7], [8]. We believe that our study refines the one published by Carmichael and colleagues since comparisons are done between perihematomal and contralateral samples of the same individuals, it provides new data of neuroreparative processes, it also confirms some of the reported findings and finally offers a robust validation and replication revision of our data demonstrating the usefulness of this type of studies to translate the results at the protein level.

Perihematomal areas have been characterized to suffer edema, apoptosis, necrosis and inflammatory processes starting early after the hemorrhagic event, peaking at days 5–6 and involving both cellular and molecular components [18], [19]. In this sense our functional analysis draws attention to several signaling pathways responsible for neuroinflammation and related genes such as *TREM-1*, *IL-8*, *IL-6*, *IL1-R1*, *CXCL2*, *CXCL5*, *CCL3*, *CCL4*, *CCL20*, *VCAM*, *ICAM* or *TLR2* among others. These genes have been found to be overexpressed in perihematomal areas related to leukocyte extravasation signaling and the explicit presence of macrophages and monocytes. IL-8 is a chemokine produced mainly by macrophages and has a pivotal role within these inflammatory and leukocyte signaling networks. To our knowledge, the only description of *IL-8* being overexpressed in perihematomal brain areas after ICH is the preceding microarray study in humans [8], although the comparison was done with control subjects. At the protein level IL-8 has never been described as a biomarker after ICH in humans. In this regard, we have previously reported IL-8 serum levels after ischemic stroke [20] and other authors have shown that patients with ischemic stroke had high numbers of *IL-8* mRNA expressing blood-mononuclear cells correlated with protein plasma level [21] and that high plasma level of IL-8 during the acute phase of ischemic stroke are maintained elevated at one week when compared to controls [22].

Interestingly, *SERPINE-1* (also known as plasminogen activator inhibitor type1), *SERPINB1* and *SERPINB9* that belong to the large superfamily of serine proteinase inhibitors, have been found overexpressed in perihematomal areas, and at least *SERPINE-1* has been also validated by quantitative RT-PCR. These proteins may affect the outcome of ICH since they inhibit serin proteases such as thrombin or tissue-type plasminogen activator which are known to exert excitotoxic effects or regulate caspase-mediated apoptosis [23], [24]. In this sense, experimental models of ICH have shown that SERPINE-1 is increased at gene and protein level around the hematoma, peaking at the first day and blocked when a specific thrombin inhibitor such as hirudin was given [25]. The authors postulate that the up-regulation of plasminogen activator inhibitor-1 may be neuroprotective by limiting thrombin or other serine protease-induced toxicity. To our knowledge, SERPINB1 and SERPINB9 have not been studied in the context of ICH or other neurological diseases. However, the protective role for SERPINB9 is proved since inhibits TNF-, ligand- and Fas ligand-induced apoptosis in certain TNF-sensitive cell lines by directly interacting with the intermediate active forms of caspase-8 and -10 [26]. In this regard, neuroserpin, another member of the serpin family has been already tested as an adjuvant therapy for tissue-type plasminogen activator administration in a rat model of embolic stroke demonstrating neuroprotective effects [27], but the therapeutic potential of SERPINE-1, SERPINB1 or SERPINB9 is still unknown.

As mentioned, several regulated genes in the perihematomal area have been related to brain repair processes such as neurogenesis, synaptic plasticity or dendrite outgrowth. Endogenous neurogenesis is known to occur under physiologic conditions in specific areas of the brain such as the subventricular zone of the lateral ventricle and the subgranular zone of the hippocampal dentate gyrus [28], [29] and after cerebral ischemia in periinfarct areas [30]. Moreover the presence of potential neural progenitor cells located in the cerebral cortex has also been reported in post-stroke murine [31] and human brain [32]. More recently some studies have reported signs of endogenous neurogenesis in perihematomal tissue after ICH. Masuda and colleagues have demonstrated that proliferation in the subventricular zone and migration towards the hemorrhage areas of neural progenitor cells occur in a collagenase-induced intracerebral hemorrhage model in rats [33]. And lately Shen and colleagues have shown that ICH induces neurogenesis in the adult human brain, since cells expressing neural stem/progenitor cell-specific markers are located in perihematomal tissue [34].

Our study proves that several pathways and specific genes which might direct brain repair processes are highly upregulated in perihematomal areas. In this regard, TGFβ, axonal guidance and actin cytoskeleton signaling are some of the canonical pathways identified in our perihematomal areas. The TGFβ superfamily has several subfamilies with numerous members: bone morphogenetic proteins (which control cell differentiation and growth), activin/inhibins (with neurotrophic and neuroprotective effects) and TGFβ's (which control proliferation, cell differentiation and apoptosis) among other molecules. Several important genes of this superfamily have been found regulated in perihematomal areas (such as *TGFβ2*, *BMP2*, *INBHA* or *TGFBI*) and further downstream signaling genes (such as *SMAD1*) indicating the potential of modulating these pathways to alter the damage induced by stroke as others have suggested [35].

Regarding axonal guidance signaling, important genes such as *LINGO-1*, expressed in neuronal tissue, part of the Nogo receptor-complex and responsible for leading the collapse of growth cone and inhibition of neurite extension [36] has been found down-regulated in perihematomal areas. The therapeutic potential of blocking LINGO-1 has been explored in other diseases of the CNS such as Parkinson's disease [37] or multiple sclerosis [38]. *PFN1*, also named profilin-1, has been found overexpressed in our study. This gene encodes for an actin-binding protein essential for neuronal differentiation and synaptic plasticity [39] but whether or no it plays an important role in neurogenesis and tissue repair after stroke still needs to be elucidated. Another example of an up-regulated gene in perihematomal tissue and potentially related with endogenous repair mechanisms is *CDC42*, a small GTPase of the Rho-subfamily. This gene is a key regulator of morphological changes in neurons responsible for dendrite outgrowth thorough dendritic cytoskeletal organization [40]. Although there are no human studies in both ICH and ischemic stroke, an experimental study in rats shows how gene therapy with hepatocyte growth factor improved significantly learning and memory during the recovery phase of stroke related to an increase of CDC42 positive pyramidal neurons in the periinfarct areas and enhanced neuritogenesis [41].

Clearly it is difficult to analyze and describe the role of the complete list of genes reported in this type of studies. We have focused our validation and replication studies on the genes that were particularly over- or underexpressed and that represent key molecules in the canonical pathways or functional networks modeled by the IPA system. However, we recognize that other genes might be of great interest too. In this regard, several genes involved in the inflammatory/anti-inflammatory networks described by Carmichael and colleagues [8] are also induced in our study (such as *CXCL2*, *CXCL3*, *IL1R1* or *ANXA2*, *TGFBI*, respectively). More specifically, 64 (13.67%) out of the 468 identified genes were also reported in this other study. Of these, 47 (73.43%) were underexpressed in both studies, 15 (23.43%) were up-regulated and only 2 (3.12%) showed divergences. Another drawback is that genes playing a central role in some of the identified pathways could not be found altered in the perihematomal areas in our study probably because the expression levels were not high or stable enough to be considered differentially expressed. This is the case of TNF which is known to be present in the brain after induced-ICH [42] or MMP9, known to be present in perihematomal areas in humans [10] and to be altered in blood related to early hematoma expansion [43] and perihematomal edema [44]. We recognize that some contamination from peripheral blood exists in our samples since brain perfusion to remove blood from brain vessels was not possible. However, we believe that this technical limitation might not be influencing our final results since comparisons have been performed with contralateral tissue of the same individuals. Finally, apparent differences in gender, alcohol consumption and presence of atrial fibrillation exist between controls and strokes in our protein study at blood level, and this could interfere with the interpretation of the results.

In conclusion, our study provides novel information with rigorous bioinformatic approaches, validation and replication data about genes and pathways that might be responsible for perihematomal secondary injury but also of endogenous repair mechanisms becoming targets of therapeutic interest.

## Supporting Information

Figure S1 **Data exploration, visualization, quality control, normalization and filtering**: **A**) RNA degradation plot, **B**) signal distribution plot, **C**) array-array intensity correlations, **D**) box-plots of the normalized data, **E**) Principal Components 2D Plot and **F**) hierarchical clustering of samples showing a cluster of all arrays. PH (perihematoma), CW (contralateral white) and CG (contralateral grey).
(TIF)

Table S1 **Group of genes identified to be Neuronal or Glial markers in healthy brain tissue and expression patterns.**
(DOC)

Table S2 **Complete list of significantly regulated genes between perihematomal and contralateral areas (n = 468).**
(DOC)

Table S3 **Ingenuity Canonical Pathways showing a significant association [-Log(Pvalue) >1.3].**
(DOC)

## References

[1] Qureshi A, Mendelow AD, Hanley DF (2009). Intracerebral Hemorrhage.. Lancet.

[2] Mayer SA, Brun NC, Begtrup K, Broderick J, Davis S (2008). Efficacy and safety of recombinant activated factor VII for acute intracerebral hemorrhage.. N Engl J Med.

[3] Mayer SA, Davis SM, Skolnick BE, Brun NC, Begtrup K (2009). Can a subset of intracerebral hemorrhage patients benefit from hemostatic therapy with recombinant activated factor VII?. Stroke.

[4] Morgenstern LB, Hemphill JC, Anderson C, Becker K, Broderick JP (2010). A Guideline for Healthcare Professionals From the American Heart Association/American Stroke Association.. Stroke.

[5] Gebel JM, Jauch EC, Brott TG, Khoury J, Sauerbeck L (2002). Natural history of perihematomal edema in patients with hyperacute spontaneous intracerebral hemorrhage.. Stroke.

[6] Inaji M, Tomita H, Tone O, Tamaki M, Suzuki R (2003). Chronological changes of perihematomal edema of human intracerebral hematoma.. Acta Neurochir Suppl.

[7] Lu A, Tang Y, Ran R, Ardizzone TL, Wagner KR (2006). Brain genomics of intracerebral hemorrhage.. J Cereb Blood Flow Metab.

[8] Carmichael ST, Vespa PM, Saver JL, Coppola G, Geschwind DH (2008). Genomic profiles of damage and protection in human intracerebral hemorrhage.. J Cereb Blood Flow Metab.

[9] Edgar R, Domrachev M, Lash AE (2002). Gene Expression Omnibus: NCBI gene expression and hybridization array data repository.. Nucleic Acids Res.

[10] Rosell A, Ortega-Aznar A, Alvarez-Sabín J, Fernández-Cadenas I, Ribó M (2006). Increased brain expression of matrix metalloproteinase-9 after ischemic and hemorrhagic human stroke.. Stroke.

[11] Sibille E, Arango V, Joeyen-Waldorf J, Wang Y, Leman S (2008). Large-scale estimates of cellular origins of mRNAs: enhancing the yield of transcriptome analyses.. J Neurosci Methods.

[12] Kim YD, Sohn NW, Kang CH, Soh Y (2002). DNA array reveals altered gene expression in response to focal cerebral ischemia.. Brain Res Bull.

[13] Raghavendra Rao VL, Bowen KK, Dhodda VK, Song GQ, Franklin JL (2002). Gene expression analysis of spontaneously hypertensive rat cerebral cortex following transient focal cerebral ischemia.. J Neurochem.

[14] Moore DF, Li H, Jeffries N, Wright V, Cooper RA (2005). Using peripheral blood mononuclear cells to determine a gene expression profile of acute ischemic stroke - A pilot investigation.. Circulation.

[15] Tang Y, Xu HC, Du XL, Lit L, Walker W (2006). Gene expression in blood changes rapidly in neutrophils and monocytes after ischemic stroke in humans: a microarray study.. J Cereb Blood Flow Metab.

[16] Vikman P, Edvinsson L (2006). Gene expression profiling in the human middle cerebral artery after cerebral ischemia.. Eur J Neurol.

[17] Mitsios N, Saka M, Krupinski J, Pennucci R, Sanfeliu C (2007). A microarray study of gene and protein regulation in human and rat brain following middle cerebral artery occlusion.. BMC Neurosci.

[18] Qureshi AI, Suri MF, Ostrow PT, Kim SH, Ali Z (2003). Apoptosis as a form of cell death in intracerebral hemorrhage.. Neurosurgery.

[19] Wang J, Doré S (2007). Inflammation after intracerebral hemorrhage.. J Cereb Blood Flow Metab.

[20] Montaner J, Rovira A, Molina CA, Arenillas JF, Ribó M (2003). Plasmatic level of neuroinflammatory markers predicts the extent of diffusion-weighted image lesions in hyperacute stroke.. J Cereb Blood Flow Metab.

[21] Kostulas N, Kivisäkk P, Huang Y, Matusevicius D, Kostulas V (1998). Ischemic stroke is associated with a systemic increase of blood mononuclear cells expressing interleukin-8 mRNA.. Stroke.

[22] Grau AJ, Reis A, Buggle F, Al-Khalaf A, Werle E (2001). Monocyte function and plasma levels of interleukin-8 in acute ischemic stroke.. J Neurol Sci.

[23] Lijnen HR (2005). Pleiotropic functions of plasminogen activator inhibitor-1.. J Thromb Haemost.

[24] Balsara RD, Ploplis VA (2008). Plasminogen activator inhibitor-1: the double-edged sword in apoptosis.. Thromb Haemost.

[25] Hua Y, Xi G, Keep RF, Wu J, Jiang Y (2002). Plasminogen activator inhibitor-1 induction after experimental intracerebral hemorrhage.. J Cereb Blood Flow Metab.

[26] Kummer JA, Micheau O, Schneider P, Bovenschen N, Broekhuizen R (2007). Ectopic expression of the serine protease inhibitor PI9 modulates death receptor-mediated apoptosis.. Cell Death Differ.

[27] Zhang Z, Zhang L, Yepes M, Jiang Q, Li Q (2002). Adjuvant treatment with neuroserpin increases the therapeutic window for tissue-type plasminogen activator administration in a rat model of embolic stroke.. Circulation.

[28] Kuhn HG, Dickinson-Anson H, Gage FH (1996). Neurogenesis in the dentate gyrus of the adult rat: age-related decrease of neuronal progenitor proliferation.. J Neurosci.

[29] Alvarez-Buylla A, Garcia-Verdugo JM (2002). Neurogenesis in adult subventricular zone.. J Neurosci.

[30] Jin K, Wang X, Xie L, Mao XO, Zhu W (2006). Evidence for stroke-induced neurogenesis in the human brain.. Proc Natl Acad Sci U S A.

[31] Nakagomi T, Taguchi A, Fujimori Y, Saino O, Nakano-Doi A (2009). Isolation and characterization of neural stem/progenitor cells from post-stroke cerebral cortex in mice.. Eur J Neurosci.

[32] Nakayama D, Matsuyama T, Ishibashi-Ueda H, Nakagomi T, Kasahara Y (2010). Injury-induced neural stem/progenitor cells in post-stroke human cerebral cortex.. Eur J Neurosci.

[33] Masuda T, Isobe Y, Aihara N, Furuyama F, Misumi S (2007). Increase in neurogenesis and neuroblast migration after a small intracerebral hemorrhage in rats.. Neurosci Lett.

[34] Shen J, Xie L, Mao X, Zhou Y, Zhan R (2008). Neurogenesis after primary intracerebral hemorrhage in adult human brain.. J Cereb Blood Flow Metab.

[35] Harvey BK, Hoffer BJ, Wang Y (2005). Stroke and TGF-beta proteins: glial cell line-derived neurotrophic factor and bone morphogenetic protein.. Pharmacol Ther.

[36] Mi S, Lee X, Shao Z, Thill G, Ji B (2004). LINGO-1 is a component of the Nogo-66 receptor/p75 signaling complex.. Nat Neurosci.

[37] Inoue H, Lin L, Lee X, Shao Z, Mendes S (2007). Inhibition of the leucine-rich repeat protein LINGO-1 enhances survival, structure, and function of dopaminergic neurons in Parkinson's disease models.. Proc Natl Acad Sci U S A.

[38] Rudick RA, Mi S, Sandrock AW (2008). LINGO-1 antagonists as therapy for multiple sclerosis: in vitro and in vivo evidence.. Expert Opin Biol Ther.

[39] Birbach A (2008). Profilin, a multi-modal regulator of neuronal plasticity.. Bioessays.

[40] Kuramoto K, Negishi M, Katoh H (2009). Regulation of dendrite growth by the Cdc42 activator Zizimin1/Dock9 in hippocampal neurons.. J Neurosci Res.

[41] Shimamura M, Sato N, Waguri S, Uchiyama Y, Hayashi T (2006). Gene transfer of hepatocyte growth factor gene improves learning and memory in the chronic stage of cerebral infarction.. Hypertension.

[42] Li ZQ, Liang GB, Xue YX, Liu YH (2009). Effects of combination treatment of dexamethasone and melatonin on brain injury in intracerebral hemorrhage model in rats.. Brain Res.

[43] Silva Y, Leira R, Tejada J, Lainez JM, Castillo J (2005). Molecular signatures of vascular injury are associated with early growth of intracerebral hemorrhage.. Stroke.

[44] Alvarez-Sabín J, Delgado P, Abilleira S, Molina CA, Arenillas J (2004). Temporal profile of matrix metalloproteinases and their inhibitors after spontaneous intracerebral hemorrhage: relationship to clinical and radiological outcome.. Stroke.

