# Supplementary figures and images for: Brain Perihematoma Genomic Profile Following Spontaneous Human Intracerebral Hemorrhage
Source: PLoS One. 2011 Feb 2;6(2):e16750. doi: 10.1371/journal.pone.0016750 (PMC3032742; doi:10.1371/journal.pone.0016750)

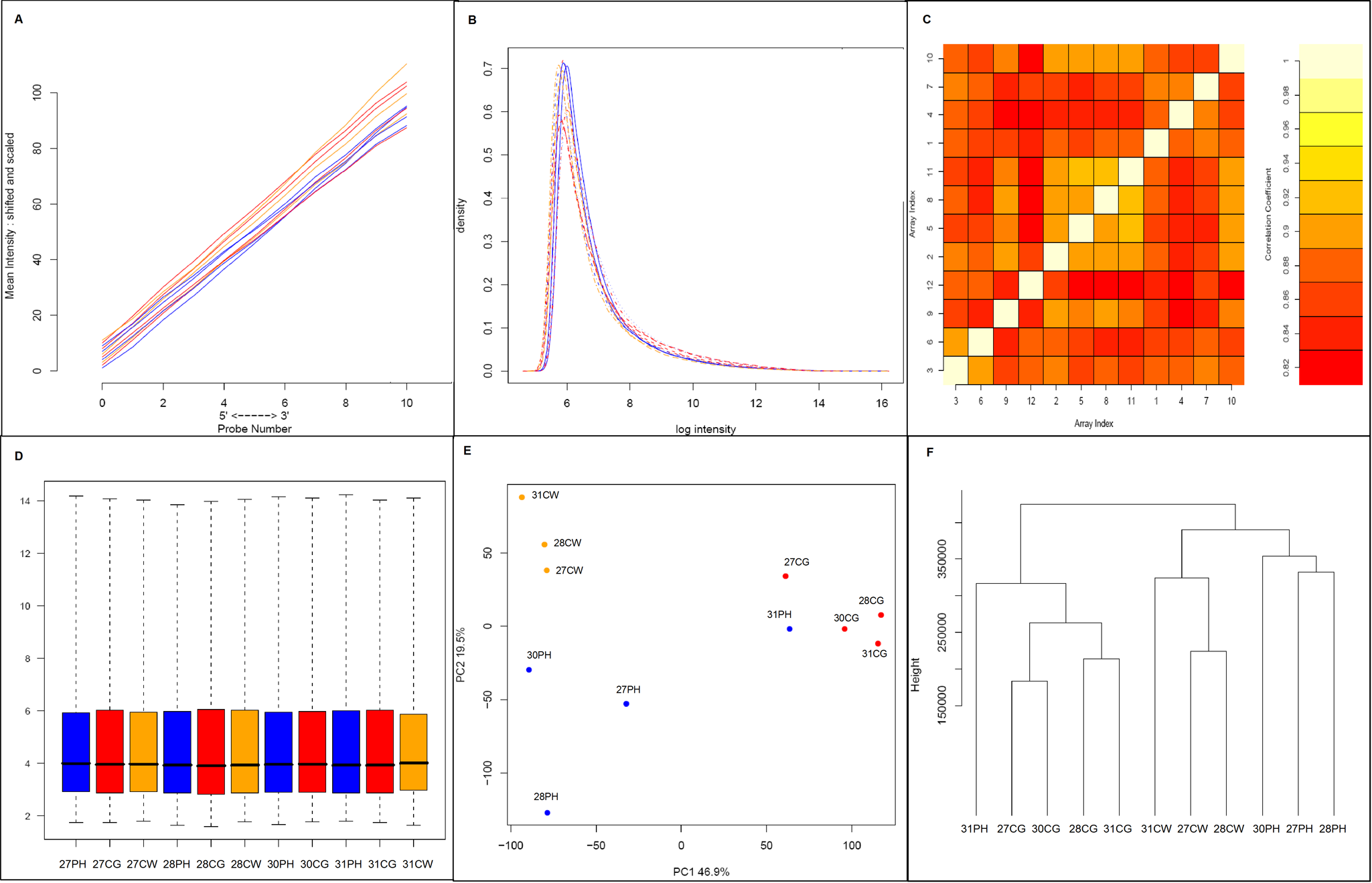

Supplement: Figure S1 — Data exploration, visualization, quality control, normalization and filtering: A) RNA degradation plot, B) signal distribution plot, C) array-array intensity correlations, D) box-plots of the normalized data, E) Principal Components 2D Plot and F) hierarchical clustering of samples showing a cluster of all arrays. PH (perihematoma), CW (contralateral white) and CG (contralateral grey). (TIF) [file pone.0016750.s001.tif]
