# Supplementary material for: Brain Perihematoma Genomic Profile Following Spontaneous Human Intracerebral Hemorrhage
Source: PLoS One. 2011 Feb 2;6(2):e16750. doi: 10.1371/journal.pone.0016750 (PMC3032742; doi:10.1371/journal.pone.0016750)
Supplement: Table S1 — Group of genes identified to be Neuronal or Glial markers in healthy brain tissue and expression patterns. (DOC) [file pone.0016750.s002.doc]

**Table S1.**

| **Symbols** | **CW vs.CG** | | | | | **PH vs.CW** | | | | | **PH vs.CG** | | | | | |
| --- | --- | --- | --- | --- | --- | --- | --- | --- | --- | --- | --- | --- | --- | --- | --- | --- |
| **Neuronal Markers** | **logFC** | **adj.P.Val** | **27** | **28** | **31** | **logFC** | **adj.P.Val** | **27** | **28** | **31** | **logFC** | **adj.P.Val** | **27** | **28** | **31** | **30** |
| CACNA1B | -4.73 | 0.0003 | -3.88 | -4.81 | -5.49 | 2.52 | 0.0502 | 2.15 | 0.94 | 4.47 | -2.80 | 0.0052 | -1.73 | -3.87 | -4.57 | -1.02 |
| CACNB2 | -4.09 | 0.0003 | -3.39 | -4.79 | -4.09 | 1 | 0.2303 | 0.56 | 0.31 | 2.14 | -3.25 | 0.0009 | -2.83 | -4.49 | -3.75 | -1.95 |
| CACNB3 | -3.47 | 0.0006 | -3.07 | -3.74 | -3.59 | 1.58 | 0.0808 | 2.32 | 0.66 | 1.77 | -2.29 | 0.0050 | -0.75 | -3.09 | -3.52 | -1.82 |
| CALB1 | -4.47 | 0.0041 | -1.23 | -6.42 | -5.76 | 1.73 | 0.2393 | 0.14 | 0.33 | 4.72 | -3.06 | 0.0179 | -1.09 | -6.09 | -4.02 | -1.04 |
| CAMK2A | -4.54 | 0.0003 | -4.10 | -4.64 | -4.88 | 1.66 | 0.0916 | 2.88 | 0.63 | 1.46 | -3.41 | 0.0013 | -1.21 | -4.02 | -5.01 | -3.42 |
| CNR1 | -6.21 | 0.0001 | -4.64 | -7.32 | -6.66 | 0.54 | 0.6221 | 1.89 | 0.15 | -0.43 | -5.73 | 0.0003 | -2.75 | -7.17 | -5.92 | -7.09 |
| GABBR2 | -3.49 | 0.0028 | -3.90 | -3.93 | -2.62 | 1.58 | 0.1502 | 2.77 | -0.36 | 2.32 | -2.46 | 0.0122 | -1.13 | -4.29 | -4.13 | -0.31 |
| GABRA1 | -5.62 | 0.0007 | -4.77 | -6.04 | -6.05 | 2.78 | 0.0680 | 2.31 | 0.35 | 5.69 | -3.68 | 0.0054 | -2.46 | -5.69 | -6.19 | -0.37 |
| GABRA2 | -3.53 | 0.0018 | -3.70 | -2.32 | -4.58 | 0.48 | 0.6424 | 1.72 | -1.99 | 1.69 | -2.75 | 0.0053 | -1.97 | -4.31 | -1.85 | -2.89 |
| GRIN2A | -4.01 | 0.0013 | -3.01 | -5.37 | -3.65 | 1.15 | 0.2853 | 0.16 | 0.43 | 2.87 | -3.43 | 0.0029 | -2.85 | -4.95 | -5.13 | -0.78 |
| SYN2 | -6.66 | 0.0001 | -5.49 | -7.85 | -6.65 | 3.49 | 0.0346 | 3.96 | 2.12 | 4.40 | -3.62 | 0.0019 | -1.53 | -5.73 | -4.97 | -2.25 |
| **Glial Markers** |  |  |  |  |  |  |  |  |  |  |  |  |  |  |  |  |
| AQP1 | 0.65 | 0.6027 | 0.040 | 1.50 | 0.40 | -2.88 | 0.0677 | -1.89 | -4.18 | -2.58 | -0.46 | 0.6651 | -1.85 | -2.68 | 4.86 | -2.18 |
| CNP | 2.44 | 0.0124 | 1.17 | 2.25 | 3.89 | -1.87 | 0.0857 | -0.68 | -3.29 | -1.63 | 1.03 | 0.1773 | 0.49 | -1.04 | 2.42 | 2.26 |
| LPAR1 | 2.73 | 0.0041 | 1.27 | 2.48 | 4.44 | -1.30 | 0.1564 | -0.87 | -1.62 | -1.40 | 1.60 | 0.0328 | 0.40 | 0.86 | 2.12 | 3.04 |
| MBP | 3.18 | 0.0039 | 1.28 | 2.64 | 5.61 | -0.66 | 0.5257 | -0.13 | -0.76 | -1.08 | 2.73 | 0.0062 | 1.15 | 1.88 | 3.38 | 4.52 |
| MOBP | 3.43 | 0.0015 | 1.77 | 3.14 | 5.39 | -1.06 | 0.2617 | -0.31 | -1.10 | -1.78 | 2.78 | 0.0038 | 1.46 | 2.04 | 4.03 | 3.61 |
| MOG | 2.49 | 0.0090 | 1.65 | 2.16 | 3.66 | -0.63 | 0.5091 | -0.96 | -1.30 | 0.36 | 2.27 | 0.0105 | 0.69 | 0.87 | 3.51 | 4.02 |
| OLIG2 | 2.01 | 0.0297 | 0.78 | 1.88 | 3.37 | -2.10 | 0.0657 | -1.50 | -3.56 | -1.25 | 0.21 | 0.7900 | -0.72 | -1.68 | 1.11 | 2.11 |

PH (perihematoma), CW (contralateral white) and CG (contralateral grey).
