# Supplementary material for: Brain Perihematoma Genomic Profile Following Spontaneous Human Intracerebral Hemorrhage
Source: PLoS One. 2011 Feb 2;6(2):e16750. doi: 10.1371/journal.pone.0016750 (PMC3032742; doi:10.1371/journal.pone.0016750)
Supplement: Table S2 — Complete list of significantly regulated genes between perihematomal and contralateral areas (n = 468). (DOC) [file pone.0016750.s003.doc]

| **Table S2.** | | | | |
| --- | --- | --- | --- | --- |
| **Symbol** | **Probe ID** | **Entrez Gene Name** | **log.Fold Change** | **Adj.P.value** |
| **ABAT** | 209460_at | 4-aminobutyrate aminotransferase | -1.80 | 0.0399 |
| **ABCC5** | 209380_s_at | ATP-binding cassette, sub-family C (CFTR/MRP), member 5 | -1.47 | 0.0445 |
| **ABLIM2** | 228132_at | actin binding LIM protein family, member 2 | -1.67 | 0.0480 |
| **ACADSB** | 226030_at | acyl-Coenzyme A dehydrogenase, short/branched chain | -1.43 | 0.0499 |
| **ADAM8** | 205180_s_at | ADAM metallopeptidase domain 8 | 2.26 | 0.0192 |
| **ADCY2** | 213217_at | adenylate cyclase 2 (brain) | -1.63 | 0.0443 |
| **ADFP** | 209122_at | adipose differentiation-related protein | 3.28 | 0.0057 |
| **ADRA1A** | 237390_at | adrenergic, alpha-1A-, receptor | -1.88 | 0.0132 |
| **AGPAT9** | 224480_s_at | 1-acylglycerol-3-phosphate O-acyltransferase 9 | 4.13 | 0.0224 |
| **AHCTF1** | 226115_at | AT hook containing transcription factor 1 | -1.73 | 0.0172 |
| **AK2** | 208967_s_at | adenylate kinase 2 | 1.74 | 0.0160 |
| **AKAP12** | 241679_at | A kinase (PRKA) anchor protein 12 | 1.31 | 0.0481 |
| **ALDH1A1** | 212224_at | aldehyde dehydrogenase 1 family, member A1 | 1.26 | 0.0396 |
| **ALOX5AP** | 204174_at | arachidonate 5-lipoxygenase-activating protein | 2.05 | 0.0148 |
| **AMACR** | 209425_at | alpha-methylacyl-CoA racemase | -1.51 | 0.0386 |
| **AMPD3** | 207992_s_at | adenosine monophosphate deaminase (isoform E) | 1.55 | 0.0266 |
| **ANKHD1** | 219081_at | ankyrin repeat and KH domain containing 1 | 2.02 | 0.0241 |
| **ANKRD43** | 230238_at | ankyrin repeat domain 43 | -2.75 | 0.0051 |
| **ANXA2** | 201590_x_at | annexin A2 | 2.89 | 0.0346 |
| **ANXA2P2** | 208816_x_at | annexin A2 pseudogene 2 | 2.43 | 0.0484 |
| **ANXA5** | 200782_at | annexin A5 | 1.76 | 0.0104 |
| **APBA2** | 209871_s_at | amyloid beta (A4) precursor protein-binding, family A, member 2 | -1.64 | 0.0319 |
| **APLP2** | 211404_s_at | amyloid beta (A4) precursor-like protein 2 | 2.10 | 0.0382 |
| **APP** | 237571_at | amyloid beta (A4) precursor protein | 2.79 | 0.0059 |
| **ARF4** | 201096_s_at | ADP-ribosylation factor 4 | 2.22 | 0.0415 |
| **ARHGAP18** | 225171_at | Rho GTPase activating protein 18 | 2.57 | 0.0346 |
| **ARHGEF4** | 205109_s_at | Rho guanine nucleotide exchange factor (GEF) 4 | -1.72 | 0.0277 |
| **ARPC5** | 1555797_a_at | actin related protein 2/3 complex, subunit 5, 16kDa | 1.52 | 0.0227 |
| **ART3** | 210147_at | ADP-ribosyltransferase 3 | -2.24 | 0.0457 |
| **ATP13A3** | 242853_at | ATPase type 13A3 | 2.39 | 0.0220 |
| **ATP1A2** | 203296_s_at | ATPase, Na+/K+ transporting, alpha 2 (+) polypeptide | -1.45 | 0.0495 |
| **ATP1B2** | 204311_at | ATPase, Na+/K+ transporting, beta 2 polypeptide | -1.71 | 0.0167 |
| **B2M** | 232311_at | beta-2-microglobulin | 2.70 | 0.0153 |
| **B3GNT5** | 225612_s_at | UDP-GlcNAc:betaGal beta-1,3-N-acetylglucosaminyltransferase 5 | 1.89 | 0.0221 |
| **BACE2** | 222446_s_at | beta-site APP-cleaving enzyme 2 | 1.77 | 0.0487 |
| **BAIAP3** | 216356_x_at | BAI1-associated protein 3 | -1.52 | 0.0399 |
| **BCAT1** | 214452_at | branched chain aminotransferase 1, cytosolic | 3.45 | 0.0006 |
| **BCL10** | 1557258_a_at | B-cell CLL/lymphoma 10 | 1.64 | 0.0188 |
| **BCL2A1** | 205681_at | BCL2-related protein A1 | 3.27 | 0.0484 |
| **BHLHB5** | 228636_at | basic helix-loop-helix domain containing, class B, 5 | -2.85 | 0.0023 |
| **BICD1** | 1554020_at | bicaudal D homolog 1 (Drosophila) | 1.43 | 0.0309 |
| **BMP2** | 205290_s_at | bone morphogenetic protein 2 | 1.35 | 0.0337 |
| **BMPR1B** | 229975_at | bone morphogenetic protein receptor, type IB | -1.92 | 0.0118 |
| **BRD4** | 239149_at | bromodomain containing 4 | 1.84 | 0.0213 |
| **BTG3** | 205548_s_at | BTG family, member 3 | 1.59 | 0.0383 |
| **BZW1L1** | 200776_s_at | basic leucine zipper and W2 domains 1 | 2.31 | 0.0346 |
| **C13orf18** | 44790_s_at | chromosome 13 open reading frame 18 | 2.80 | 0.0346 |
| **C14orf28** | 235369_at | chromosome 14 open reading frame 28 | -1.58 | 0.0268 |
| **C15orf48** | 223484_at | chromosome 15 open reading frame 48 | 4.38 | 0.0046 |
| **C18orf1** | 207996_s_at | chromosome 18 open reading frame 1 | -1.94 | 0.0208 |
| **C18orf19** | 235022_at | chromosome 18 open reading frame 19 | 1.58 | 0.0229 |
| **C1orf79** | 223774_at | small nucleolar RNA host gene 12 | 2.21 | 0.0172 |
| **C20orf177** | 225313_at | chromosome 20 open reading frame 177 | -2.12 | 0.0108 |
| **C21orf86** | 228909_at | chromosome 21 open reading frame 86 | -1.64 | 0.0331 |
| **C5AR1** | 220088_at | complement component 5a receptor 1 | 2.54 | 0.0039 |
| **C5orf35** | 238465_at | chromosome 5 open reading frame 35 | 1.27 | 0.0406 |
| **C5orf41** | 235556_at | chromosome 5 open reading frame 41 | -1.69 | 0.0193 |
| **C7orf44** | 241410_at | chromosome 7 open reading frame 44 | -1.62 | 0.0195 |
| **C9orf19** | 225604_s_at | GLI pathogenesis-related 2 | 2.26 | 0.0069 |
| **CA2** | 209301_at | carbonic anhydrase II | 1.43 | 0.0294 |
| **CADM1** | 209031_at | cell adhesion molecule 1 | -1.68 | 0.0166 |
| **CALN1** | 230698_at | calneuron 1 | -2.31 | 0.0229 |
| **CAMTA1** | 213268_at | calmodulin binding transcription activator 1 | -1.84 | 0.0267 |
| **CAST** | 207467_x_at | calpastatin | 1.87 | 0.0146 |
| **CAV1** | 212097_at | caveolin 1, caveolae protein, 22kDa | 1.62 | 0.0474 |
| **CAV2** | 203324_s_at | caveolin 2 | 1.81 | 0.0296 |
| **CBX7** | 212914_at | chromobox homolog 7 | -1.57 | 0.0323 |
| **CCDC126** | 228087_at | coiled-coil domain containing 126 | -1.58 | 0.0446 |
| **CCL20** | 205476_at | chemokine (C-C motif) ligand 20 | 4.81 | 0.0051 |
| **CCL3** | 205114_s_at | chemokine (C-C motif) ligand 3 | 3.29 | 0.0224 |
| **CCL4** | 204103_at | chemokine (C-C motif) ligand 4 | 2.76 | 0.0304 |
| **CCNL1** | 220046_s_at | cyclin L1 | 1.32 | 0.0383 |
| **CD163** | 215049_x_at | CD163 molecule | 4.16 | 0.0379 |
| **CD24** | 216379_x_at | CD24 molecule | -1.33 | 0.0353 |
| **CD300A** | 209933_s_at | CD300a molecule | 1.78 | 0.0319 |
| **CD44** | 204490_s_at | CD44 molecule (Indian blood group) | 2.13 | 0.0171 |
| **CD47** | 243888_at | CD47 molecule | 2.32 | 0.0144 |
| **CD55** | 1555950_a_at | CD55 molecule, decay accelerating factor for complement (Cromer blood group) | 2.48 | 0.0056 |
| **CD59** | 200984_s_at | CD59 molecule, complement regulatory protein | 2.36 | 0.0410 |
| **CD69** | 209795_at | CD69 molecule | 2.07 | 0.0167 |
| **CD9** | 201005_at | CD9 molecule | 1.51 | 0.0210 |
| **CD93** | 202878_s_at | CD93 molecule | 2.38 | 0.0313 |
| **CDC14B** | 230887_at | CDC14 cell division cycle 14 homolog B | 1.94 | 0.0157 |
| **CDC42** | 208727_s_at | cell division cycle 42 (GTP binding protein. 25kDa) | 1.61 | 0.0317 |
| **CDKL2** | 236331_at | cyclin-dependent kinase-like 2 (CDC2-related kinase) | -1.53 | 0.0434 |
| **CDV3** | 213554_s_at | CDV3 homolog (mouse) | 1.71 | 0.0137 |
| **CFLAR** | 210563_x_at | CASP8 and FADD-like apoptosis regulator | 1.80 | 0.0184 |
| **CHD2** | 1554014_at | chromodomain helicase DNA binding protein 2 | 2.14 | 0.0163 |
| **CHD6** | 225031_at | chromodomain helicase DNA binding protein 6 | -1.79 | 0.0165 |
| **CLASP1** | 212752_at | cytoplasmic linker associated protein 1 | -1.53 | 0.0466 |
| **CLEC2B** | 209732_at | C-type lectin domain family 2, member B | 2.53 | 0.0221 |
| **CLIC1** | 208659_at | chloride intracellular channel 1 | 1.87 | 0.0477 |
| **CLIC4** | 201559_s_at | chloride intracellular channel 4 | 2.36 | 0.0105 |
| **CLU** | 1559228_at | clusterin | 2.12 | 0.0197 |
| **CMTM7** | 226017_at | CKLF-like MARVEL transmembrane domain containing 7 | 1.36 | 0.0450 |
| **CNDP1** | 223699_at | carnosine dipeptidase 1 (metallopeptidase M20 family) | 2.17 | 0.0341 |
| **COL1A2** | 202403_s_at | collagen, type I, alpha 2 | 2.32 | 0.0360 |
| **COL6A3** | 201438_at | collagen, type VI, alpha 3 | 1.55 | 0.0405 |
| **CORO1C** | 221676_s_at | coronin, actin binding protein, 1C | 1.42 | 0.0367 |
| **COTL1** | 1556346_at | coactosin-like 1 | 1.56 | 0.0236 |
| **CRB1** | 244403_at | crumbs homolog 1 | -2.31 | 0.0039 |
| **CREBBP** | 228177_at | CREB binding protein | -1.60 | 0.0187 |
| **CRLF3** | 235803_at | cytokine receptor-like factor 3 | -1.80 | 0.0112 |
| **CSPG5** | 39966_at | chondroitin sulfate proteoglycan 5 (neuroglycan C) | -1.87 | 0.0202 |
| **CTNNB1** | 242558_at | catenin (cadherin-associated protein), beta 1 | 1.69 | 0.0336 |
| **CTSL1** | 202087_s_at | cathepsin L1 | 1.85 | 0.0145 |
| **CTTN** | 214073_at | cortactin | 2.71 | 0.0043 |
| **CUGBP2** | 227178_at | CUG triplet repeat, RNA binding protein 2 | -2.79 | 0.0016 |
| **CX3CR1** | 205898_at | chemokine (C-X3-C motif) receptor 1 | -2.11 | 0.0392 |
| **CXCL1** | 204470_at | chemokine (C-X-C motif) ligand 1 (melanoma growth stimulating activity, alpha) | 4.01 | 0.0037 |
| **CXCL16** | 223454_at | chemokine (C-X-C motif) ligand 16 | 1.89 | 0.0193 |
| **CXCL2** | 209774_x_at | chemokine (C-X-C motif) ligand 2 | 3.82 | 0.0133 |
| **CXCL3** | 207850_at | chemokine (C-X-C motif) ligand 3 | 3.66 | 0.0114 |
| **CXCL5** | 214974_x_at | chemokine (C-X-C motif) ligand 5 | 4.45 | 0.0346 |
| **CYLD** | 222142_at | cylindromatosis (turban tumor syndrome) | 2.33 | 0.0030 |
| **CYR61** | 210764_s_at | cysteine-rich, angiogenic inducer, 61 | 1.66 | 0.0399 |
| **DCN** | 209335_at | decorin | 2.25 | 0.0347 |
| **DDIT3** | 209383_at | DNA-damage-inducible transcript 3 | 1.66 | 0.0161 |
| **DNAJA4** | 1554334_a_at | DnaJ (Hsp40) homolog, subfamily A, member 4 | 2.98 | 0.0346 |
| **DNAJB1** | 200664_s_at | DnaJ (Hsp40) homolog, subfamily B, member 1 | 2.83 | 0.0097 |
| **DOCK1** | 237311_at | dedicator of cytokinesis 1 | 1.46 | 0.0286 |
| **DPP6** | 228546_at | dipeptidyl-peptidase 6 | -1.92 | 0.0242 |
| **DTNA** | 244142_at | dystrobrevin, alpha | -2.06 | 0.0130 |
| **DUSP5** | 209457_at | dual specificity phosphatase 5 | 2.85 | 0.0347 |
| **EDN1** | 222802_at | endothelin 1 | 1.91 | 0.0494 |
| **EEF2K** | 225546_at | eukaryotic elongation factor-2 kinase | -1.62 | 0.0178 |
| **EFHA2** | 238458_at | EF-hand domain family, member A2 | -1.73 | 0.0498 |
| **EGR1** | 201693_s_at | early growth response 1 | 1.99 | 0.0273 |
| **EIF4A1** | 214805_at | eukaryotic translation initiation factor 4A, isoform 1 | 1.97 | 0.0175 |
| **EIF4EBP1** | 231069_at | eukaryotic translation initiation factor 4E binding protein 1 | -1.69 | 0.0140 |
| **ELF4** | 31845_at | E74-like factor 4 (ets domain transcription factor) | 1.58 | 0.0331 |
| **ELK3** | 221773_at | ELK3, ETS-domain protein (SRF accessory protein 2) | 2.25 | 0.0457 |
| **ELL2** | 240038_at | elongation factor, RNA polymerase II, 2 | 2.65 | 0.0072 |
| **EMP1** | 201325_s_at | epithelial membrane protein 1 | 1.77 | 0.0493 |
| **EMX2** | 221950_at | empty spiracles homeobox 2 | -1.63 | 0.0222 |
| **ERBB4** | 214053_at | v-erb-a erythroblastic leukemia viral oncogene homolog 4 (avian) | -2.33 | 0.0098 |
| **ERCC4** | 235215_at | excision repair cross-complementing rodent repair deficiency, complementation group 4 | -1.78 | 0.0225 |
| **ERO1L** | 222646_s_at | ERO1-like (S. cerevisiae) | 2.82 | 0.0044 |
| **ETS1** | 224833_at | v-ets erythroblastosis virus E26 oncogene homolog 1 (avian) | 1.69 | 0.0269 |
| **ETS2** | 201329_s_at | v-ets erythroblastosis virus E26 oncogene homolog 2 (avian) | 2.26 | 0.0472 |
| **EVI2B** | 211742_s_at | ecotropic viral integration site 2B | 1.55 | 0.0307 |
| **EXT1** | 230183_at | exostoses (multiple) 1 | -1.84 | 0.0134 |
| **F11R** | 224097_s_at | F11 receptor | 1.56 | 0.0431 |
| **FAM133A** | 231131_at | family with sequence similarity 133, member A | -1.82 | 0.0144 |
| **FAM49A** | 208092_s_at | family with sequence similarity 49, member A | 1.95 | 0.0457 |
| **FARP1** | 239246_at | FERM, RhoGEF (ARHGEF) and pleckstrin domain protein 1 (chondrocyte-derived) | -1.62 | 0.0312 |
| **FBXL18** | 227500_at | F-box and leucine-rich repeat protein 18 | -1.51 | 0.0369 |
| **FBXO31** | 224162_s_at | F-box protein 31 | -1.72 | 0.0123 |
| **FBXO33** | 226970_at | F-box protein 33 | -1.63 | 0.0266 |
| **FCER1G** | 204232_at | Fc fragment of IgE, high affinity I, receptor for; gamma polypeptide | 1.89 | 0.0319 |
| **FGD3** | 227811_at | FYVE, RhoGEF and PH domain containing 3 | 1.55 | 0.0361 |
| **FGF2** | 204422_s_at | fibroblast growth factor 2 (basic) | -1.53 | 0.0304 |
| **FGFR1OP2** | 1556283_s_at | FGFR1 oncogene partner 2 | 2.99 | 0.0026 |
| **FGFR3** | 204379_s_at | fibroblast growth factor receptor 3 | -2.05 | 0.0319 |
| **FGR** | 208438_s_at | Gardner-Rasheed feline sarcoma viral (v-fgr) oncogene homolog | 2.11 | 0.0169 |
| **FIGN** | 239710_at | fidgetin | -1.57 | 0.0172 |
| **FKBP1A** | 210187_at | FK506 binding protein 1A, 12kDa | 1.65 | 0.0359 |
| **FLJ90757** | 1566557_at | hypothetical protein LOC440465 | -2.05 | 0.0149 |
| **FNIP2** | 243675_at | folliculin interacting protein 2 | 1.81 | 0.0317 |
| **FOSL2** | 241824_at | FOS-like antigen 2 | 1.88 | 0.0149 |
| **FOXG1** | 206018_at | forkhead box G1 | -3.75 | 0.0193 |
| **FRMD4A** | 225163_at | FERM domain containing 4A | -1.64 | 0.0356 |
| **FUT9** | 214046_at | fucosyltransferase 9 (alpha (1,3) fucosyltransferase) | -1.92 | 0.0219 |
| **FXYD6** | 217897_at | FXYD domain containing ion transport regulator 6 | -1.88 | 0.0190 |
| **G0S2** | 213524_s_at | G0/G1switch 2 | 3.87 | 0.0021 |
| **GLCCI1** | 225706_at | glucocorticoid induced transcript 1 | -1.87 | 0.0103 |
| **GLCE** | 213552_at | glucuronic acid epimerase | -1.52 | 0.0279 |
| **GLT25D2** | 237382_at | glycosyltransferase 25 domain containing 2 | -2.83 | 0.0457 |
| **GLUL** | 200648_s_at | glutamate-ammonia ligase (glutamine synthetase) | 1.69 | 0.0383 |
| **GMFB** | 202543_s_at | glia maturation factor, beta | 1.65 | 0.0225 |
| **GPHN** | 223319_at | gephyrin | -1.56 | 0.0295 |
| **GPRC5A** | 203108_at | G protein-coupled receptor, family C, group 5, member A | 3.45 | 0.0047 |
| **GRIN2C** | 210400_at | glutamate receptor, ionotropic, N-methyl D-aspartate 2C | -1.55 | 0.0288 |
| **GRLF1** | 229397_s_at | glucocorticoid receptor DNA binding factor 1 | -1.61 | 0.0284 |
| **HBA1** | 217414_x_at | hemoglobin, alpha 1 | 2.20 | 0.0168 |
| **HBA2** | 211745_x_at | hemoglobin, alpha 2 | 2.47 | 0.0127 |
| **HBB** | 209116_x_at | hemoglobin, beta | 3.39 | 0.0472 |
| **HBD** | 206834_at | hemoglobin, delta | 2.67 | 0.0112 |
| **HBG1** | 204848_x_at | hemoglobin, gamma A | 2.18 | 0.0162 |
| **HBG2** | 213515_x_at | hemoglobin, gamma G | 3.10 | 0.0088 |
| **HEBP2** | 203430_at | heme binding protein 2 | 2.47 | 0.0382 |
| **HIST2H2AA3** | 218280_x_at | histone cluster 2, H2aa3 | 2.18 | 0.0144 |
| **HIVEP3** | 235122_at | human immunodeficiency virus type I enhancer binding protein 3 | -1.96 | 0.0183 |
| **HK2** | 202934_at | hexokinase 2 | 2.18 | 0.0038 |
| **HLA-B** | 209140_x_at | major histocompatibility complex, class I, B | 2.15 | 0.0457 |
| **HLA-C** | 214459_x_at | major histocompatibility complex, class I, C | 1.93 | 0.0484 |
| **HLA-DQB1** | 211654_x_at | major histocompatibility complex, class II, DQ beta 1 | 1.47 | 0.0401 |
| **HMOX1** | 203665_at | heme oxygenase (decycling) 1 | 2.31 | 0.0147 |
| **HNRPD** | 236000_s_at | heterogeneous nuclear ribonucleoprotein D | 1.44 | 0.0409 |
| **HNRPM** | 1555843_at | heterogeneous nuclear ribonucleoprotein M | 1.40 | 0.0400 |
| **HSP90B1** | 216449_x_at | heat shock protein 90kDa beta (Grp94), member 1 | 1.45 | 0.0360 |
| **HSPA5** | 211936_at | heat shock 70kDa protein 5 (glucose-regulated protein, 78kDa) | 1.64 | 0.0315 |
| **HSPA6** | 213418_at | heat shock 70kDa protein 6 (HSP70B') | 3.56 | 0.0044 |
| **HSPD1** | 200806_s_at | heat shock 60kDa protein 1 (chaperonin) | 1.75 | 0.0121 |
| **ICAM1** | 202637_s_at | intercellular adhesion molecule 1 | 1.79 | 0.0310 |
| **ID2** | 213931_at | inhibitor of DNA binding 2, dominant negative helix-loop-helix protein | 2.32 | 0.0484 |
| **IFI30** | 201422_at | interferon, gamma-inducible protein 30 | 2.05 | 0.0174 |
| **IFRD1** | 202147_s_at | interferon-related developmental regulator 1 | 2.18 | 0.0043 |
| **IGF2** | 202409_at | insulin-like growth factor 2 (somatomedin A) | 1.42 | 0.0229 |
| **IKZF2** | 231929_at | IKAROS family zinc finger 2 (Helios) | -2.19 | 0.0484 |
| **IL18RAP** | 207072_at | interleukin 18 receptor accessory protein | 2.87 | 0.0104 |
| **IL1R1** | 202948_at | interleukin 1 receptor, type I | 2.76 | 0.0348 |
| **IL1RN** | 212657_s_at | interleukin 1 receptor antagonist | 3.30 | 0.0457 |
| **IL6** | 205207_at | interleukin 6 (interferon, beta 2) | 2.48 | 0.0286 |
| **IL8** | 202859_x_at | interleukin 8 | 5.16 | 0.0084 |
| **INF2** | 224469_s_at | inverted formin, FH2 and WH2 domain containing | 1.83 | 0.0162 |
| **INHBA** | 210511_s_at | inhibin, beta A | 3.03 | 0.0472 |
| **INSIG1** | 201627_s_at | insulin induced gene 1 | 1.47 | 0.0230 |
| **IRAK2** | 231779_at | interleukin-1 receptor-associated kinase 2 | 1.33 | 0.0392 |
| **ISG20** | 204698_at | interferon stimulated exonuclease gene 20kDa | 2.79 | 0.0037 |
| **ITGA5** | 201389_at | integrin, alpha 5 (fibronectin receptor, alpha polypeptide) | 1.52 | 0.0280 |
| **ITGAM** | 205786_s_at | integrin, alpha M (complement component 3 receptor 3 subunit) | 1.60 | 0.0382 |
| **ITGAX** | 210184_at | integrin, alpha X (complement component 3 receptor 4 subunit) | 2.07 | 0.0063 |
| **ITGB1** | 1553530_a_at | integrin, beta 1 (fibronectin receptor, beta polypeptide, antigen CD29 includes MDF2, MSK12) | 2.98 | 0.0027 |
| **ITPKB** | 235213_at | inositol 1,4,5-trisphosphate 3-kinase B | 1.88 | 0.0220 |
| **IVNS1ABP** | 206245_s_at | influenza virus NS1A binding protein | 1.85 | 0.0132 |
| **JAKMIP3** | 233076_at | janus kinase and microtubule interacting protein 3 | -2.00 | 0.0472 |
| **JMJD6** | 212723_at | jumonji domain containing 6 | 1.46 | 0.0403 |
| **KALRN** | 227750_at | kalirin, RhoGEF kinase | -1.44 | 0.0288 |
| **KBTBD6** | 226479_at | kelch repeat and BTB (POZ) domain containing 6 | -1.72 | 0.0222 |
| **KBTBD7** | 229970_at | kelch repeat and BTB (POZ) domain containing 7 | -1.93 | 0.0204 |
| **KCNAB1** | 210078_s_at | potassium voltage-gated channel, shaker-related subfamily, beta member 1 | -2.20 | 0.0142 |
| **KDELR2** | 200700_s_at | KDEL (Lys-Asp-Glu-Leu) endoplasmic reticulum protein retention receptor 2 | 1.62 | 0.0375 |
| **KIAA0232** | 232366_at | KIAA0232 | -2.21 | 0.0144 |
| **KIAA0802** | 213358_at | KIAA0802 | -2.34 | 0.0065 |
| **KIAA1600** | 226155_at | family with sequence similarity 160, member B1 | -1.45 | 0.0375 |
| **KIRREL3** | 240402_at | kin of IRRE like 3 (Drosophila) | -2.36 | 0.0379 |
| **KLF4** | 221841_s_at | Kruppel-like factor 4 (gut) | 2.95 | 0.0379 |
| **KLHL7** | 220239_at | kelch-like 7 (Drosophila) | 1.98 | 0.0268 |
| **LAPTM5** | 201720_s_at | lysosomal multispanning membrane protein 5 | 2.45 | 0.0197 |
| **LEF1** | 221558_s_at | lymphoid enhancer-binding factor 1 | 1.47 | 0.0307 |
| **LEF1** | 221558_s_at | lymphoid enhancer-binding factor 1 | 2.35 | 0.0379 |
| **LHX2** | 206140_at | LIM homeobox 2 | -3.74 | 0.0079 |
| **LINGO1** | 227933_at | leucine rich repeat and Ig domain containing 1 | -1.66 | 0.0229 |
| **LMCD1** | 218574_s_at | LIM and cysteine-rich domains 1 | 1.52 | 0.0319 |
| **LMNA** | 203411_s_at | lamin A/C | 1.78 | 0.0141 |
| **LOC144874** | 1566257_at | hypothetical protein LOC144874 | -1.76 | 0.0195 |
| **LOC153364** | 230298_at | similar to metallo-beta-lactamase superfamily protein | -1.64 | 0.0258 |
| **LOC203274** | 232034_at | hypothetical protein LOC203274 | -1.66 | 0.0146 |
| **LOC283588** | 1557113_at | hypothetical protein LOC283588 | -2.24 | 0.0472 |
| **LOC283713** | 230783_at | hypothetical protein LOC283713 | -1.76 | 0.0290 |
| **LOC284801** | 225767_at | hypothetical protein LOC284801 | 3.15 | 0.0040 |
| **LOC285147** | 242852_at | hypothetical protein LOC285147 | -2.05 | 0.0188 |
| **LOC541471** | 225799_at | hypothetical LOC541471 | 3.21 | 0.0346 |
| **LOC54492** | 225355_at | hypothetical LOC54492 | -1.73 | 0.0147 |
| **LOC653506** | 225955_at | similar to meteorin, glial cell differentiation regulator-like | 2.82 | 0.0346 |
| **LOC727773** | 228287_at | similar to p28 ING5 | -1.42 | 0.0443 |
| **LOC729436** | 227866_at | hypothetical LOC729436 | -1.54 | 0.0286 |
| **LOC729446** | 238043_at | hypothetical LOC729446 | -2.33 | 0.0035 |
| **LOC730631** | 230738_at | hypothetical LOC730631 | -1.37 | 0.0405 |
| **LOC91461** | 225380_at | protein kinase domain containing, cytoplasmic homolog (mouse) | -1.32 | 0.0437 |
| **LRRC8B** | 212976_at | leucine rich repeat containing 8 family, member B | -2.27 | 0.0027 |
| **LRRC8C** | 223533_at | leucine rich repeat containing 8 family, member C | 1.40 | 0.0357 |
| **LYZ** | 213975_s_at | lysozyme (renal amyloidosis) | 2.85 | 0.0202 |
| **LZIC** | 226087_at | leucine zipper and CTNNBIP1 domain containing | 1.47 | 0.0343 |
| **MAFF** | 205193_at | v-maf musculoaponeurotic fibrosarcoma oncogene homolog F (avian) | 1.92 | 0.0128 |
| **MAN1C1** | 218918_at | mannosidase, alpha, class 1C, member 1 | -2.07 | 0.0145 |
| **MAP2K3** | 215498_s_at | mitogen-activated protein kinase kinase 3 | 1.72 | 0.0235 |
| **MAPK8** | 229664_at | mitogen-activated protein kinase 8 | -1.66 | 0.0225 |
| **MAPRE1** | 200712_s_at | microtubule-associated protein, RP/EB family, member 1 | 1.61 | 0.0265 |
| **MARCO** | 205819_at | macrophage receptor with collagenous structure | 2.17 | 0.0478 |
| **MAST3** | 213045_at | microtubule associated serine/threonine kinase 3 | -2.03 | 0.0071 |
| **MED28** | 218438_s_at | mediator complex subunit 28 | 1.30 | 0.0436 |
| **MEF2D** | 225641_at | myocyte enhancer factor 2D | -1.63 | 0.0229 |
| **MGC24039** | 228551_at | DENN/MADD domain containing 5B | -1.58 | 0.0359 |
| **MGC3032** | 218641_at | hypothetical protein MGC3032 | -1.63 | 0.0351 |
| **MPZL2** | 203780_at | myelin protein zero-like 2 | 2.43 | 0.0457 |
| **MSR1** | 214770_at | macrophage scavenger receptor 1 | 2.01 | 0.0494 |
| **MUM1L1** | 229160_at | melanoma associated antigen (mutated) 1-like 1 | -2.82 | 0.0014 |
| **MXD1** | 228846_at | MAX dimerization protein 1 | 2.18 | 0.0192 |
| **MYCT1** | 231947_at | myc target 1 | 2.69 | 0.0379 |
| **NAMPT** | 236495_at | nicotinamide phosphoribosyltransferase | 4.02 | 0.0082 |
| **NANP** | 228073_at | N-acetylneuraminic acid phosphatase | -1.48 | 0.0291 |
| **NARG2** | 242639_at | NMDA receptor regulated 2 | 1.74 | 0.0104 |
| **NCAM1** | 214952_at | neural cell adhesion molecule 1 | 1.36 | 0.0419 |
| **NCF2** | 209949_at | neutrophil cytosolic factor 2 | 2.60 | 0.0116 |
| **NDP** | 206022_at | Norrie disease (pseudoglioma) | -1.88 | 0.0156 |
| **NEDD9** | 1569020_at | neural precursor cell expressed, developmentally down-regulated 9 | 2.18 | 0.0125 |
| **NFKBIA** | 201502_s_at | nuclear factor of kappa light polypeptide gene enhancer in B-cells inhibitor, alpha | 1.81 | 0.0251 |
| **NFKBIZ** | 223217_s_at | nuclear factor of kappa light polypeptide gene enhancer in B-cells inhibitor, zeta | 1.62 | 0.0408 |
| **NIPBL** | 242352_at | Nipped-B homolog (Drosophila) | 1.83 | 0.0201 |
| **NP** | 201695_s_at | nucleoside phosphorylase | 2.89 | 0.0472 |
| **NR2F1** | 209505_at | nuclear receptor subfamily 2, group F, member 1 | -1.36 | 0.0443 |
| **NR3C2** | 205259_at | nuclear receptor subfamily 3, group C, member 2 | -1.74 | 0.0277 |
| **NR4A2** | 235739_at | nuclear receptor subfamily 4, group A, member 2 | 1.71 | 0.0303 |
| **NTRK2** | 229463_at | neurotrophic tyrosine kinase, receptor, type 2 | -2.54 | 0.0049 |
| **NUAK1** | 204589_at | NUAK family, SNF1-like kinase, 1 | -1.68 | 0.0162 |
| **NUDT4** | 206302_s_at | nudix (nucleoside diphosphate linked moiety X)-type motif 4 | 2.89 | 0.0415 |
| **NUPL1** | 204435_at | nucleoporin like 1 | 2.45 | 0.0347 |
| **OBFC2A** | 233085_s_at | oligonucleotide/oligosaccharide-binding fold containing 2A | 2.22 | 0.0417 |
| **OBSL1** | 212775_at | obscurin-like 1 | -1.40 | 0.0362 |
| **OCIAD2** | 225314_at | OCIA domain containing 2 | 2.13 | 0.0381 |
| **ODZ4** | 213273_at | odz, odd Oz/ten-m homolog 4 (Drosophila) | -1.80 | 0.0244 |
| **OLR1** | 210004_at | oxidized low density lipoprotein (lectin-like) receptor 1 | 2.24 | 0.0211 |
| **ORMDL2** | 218556_at | ORM1-like 2 (S. cerevisiae) | 1.73 | 0.0193 |
| **PANK1** | 226649_at | pantothenate kinase 1 | -1.85 | 0.0324 |
| **PAPLN** | 226435_at | papilin, proteoglycan-like sulfated glycoprotein | -2.03 | 0.0212 |
| **PAPOLG** | 222273_at | poly(A) polymerase gamma | -1.55 | 0.0222 |
| **PCDHA12** | 210674_s_at | protocadherin alpha 12 | -1.69 | 0.0273 |
| **PCYOX1** | 225274_at | prenylcysteine oxidase 1 | -1.61 | 0.0288 |
| **PDE4D** | 204491_at | phosphodiesterase 4D, cAMP-specific (phosphodiesterase E3 dunce homolog, Drosophila) | -1.81 | 0.0229 |
| **PDLIM1** | 208690_s_at | PDZ and LIM domain 1 | 2.47 | 0.0457 |
| **PDZD2** | 209493_at | PDZ domain containing 2 | -1.90 | 0.0451 |
| **PFN1** | 200634_at | profilin 1 | 1.49 | 0.0364 |
| **PHLDA2** | 209803_s_at | pleckstrin homology-like domain, family A, member 2 | 2.85 | 0.0347 |
| **PLAU** | 205479_s_at | plasminogen activator, urokinase | 2.51 | 0.0458 |
| **PLAUR** | 210845_s_at | plasminogen activator, urokinase receptor | 3.27 | 0.0374 |
| **PLEKHA6** | 229245_at | pleckstrin homology domain containing. family A member 6 | -2.18 | 0.0108 |
| **PPARGC1A** | 219195_at | peroxisome proliferator-activated receptor gamma, coactivator 1 alpha | -2.38 | 0.0248 |
| **PPIL4** | 1558445_at | peptidylprolyl isomerase (cyclophilin)-like 4 | -2,01 | 0.0077 |
| **PPM1K** | 235061_at | protein phosphatase 1K (PP2C domain containing) | -1.57 | 0.0309 |
| **PPP1R15A** | 37028_at | protein phosphatase 1, regulatory (inhibitor) subunit 15A | 1.53 | 0.0178 |
| **PPP1R3B** | 222662_at | protein phosphatase 1, regulatory (inhibitor) subunit 3B | 1.46 | 0.0386 |
| **PRAGMIN** | 235085_at | homolog of rat pragma of Rnd2 | -2.37 | 0.0062 |
| **PRC1** | 218009_s_at | protein regulator of cytokinesis 1 | -1.81 | 0.0135 |
| **PRDM16** | 232424_at | PR domain containing 16 | -2.72 | 0.0134 |
| **PRKCH** | 218764_at | protein kinase C, eta | 2.86 | 0.0346 |
| **PRRT2** | 227192_at | proline-rich transmembrane protein 2 | -2.90 | 0.0110 |
| **PSCDBP** | 209606_at | cytohesin 1 interacting protein | 3.51 | 0.0112 |
| **PSME4** | 212219_at | proteasome (prosome, macropain) activator subunit 4 | 1.82 | 0.0132 |
| **PSMG1** | 238890_at | proteasome (prosome, macropain) assembly chaperone 1 | -1.99 | 0.0148 |
| **PTCH1** | 209815_at | patched homolog 1 (Drosophila) | -1.88 | 0.0163 |
| **PTGER4** | 204897_at | prostaglandin E receptor 4 (subtype EP4) | 2.25 | 0.0230 |
| **PTGS1** | 215813_s_at | prostaglandin-endoperoxide synthase 1 (prostaglandin G/H synthase and cyclooxygenase) | 2.18 | 0.0124 |
| **PTK2** | 241453_at | PTK2 protein tyrosine kinase 2 | 2.42 | 0.0051 |
| **PTP4A1** | 200730_s_at | protein tyrosine phosphatase type IVA, member 1 | 2.31 | 0.0031 |
| **PTPRC** | 212587_s_at | protein tyrosine phosphatise, receptor type, C | 2.24 | 0.0221 |
| **PTX3** | 206157_at | pentraxin-related gene, rapidly induced by IL-1 beta | 3.25 | 0.0171 |
| **RAB27A** | 209514_s_at | RAB27A, member RAS oncogene family | 1.76 | 0.0253 |
| **RAC2** | 213603_s_at | ras-related C3 botulinum toxin substrate 2 (rho family, small GTP binding protein Rac2) | 1.86 | 0.0474 |
| **RALA** | 214435_x_at | v-ral simian leukemia viral oncogene homolog A (ras related) | 1.80 | 0.0202 |
| **RALGPS1** | 204199_at | Ral GEF with PH domain and SH3 binding motif 1 | -1.79 | 0.0192 |
| **RASGEF1A** | 230563_at | RasGEF domain family, member 1A | -1.95 | 0.0198 |
| **RASGRP3** | 205801_s_at | RAS guanyl releasing protein 3 (calcium and DAG-regulated) | 2.88 | 0.0037 |
| **RASSF3** | 227167_s_at | Ras association (RalGDS/AF-6) domain family member 3 | 2.56 | 0.0346 |
| **RBM22** | 222527_s_at | RNA binding motif protein 22 | 1.80 | 0.0184 |
| **RBMS1** | 225265_at | RNA binding motif, single stranded interacting protein 1 | 1.37 | 0.0401 |
| **RELN** | 205923_at | reelin | -1.29 | 0.0372 |
| **RFX4** | 223673_at | regulatory factor X, 4 (influences HLA class II expression) | -1.55 | 0.0284 |
| **RGS1** | 202988_s_at | regulator of G-protein signaling 1 | 3.87 | 0.0030 |
| **RGS16** | 209324_s_at | regulator of G-protein signaling 16 | 2.19 | 0.0043 |
| **RGS2** | 202388_at | regulator of G-protein signaling 2, 24kDa | 1.35 | 0.0341 |
| **RGS3** | 203823_at | regulator of G-protein signaling 3 | 1.41 | 0.0449 |
| **RIPK5** | 229017_s_at | receptor interacting protein kinase 5 | -1.58 | 0.0254 |
| **RIT1** | 209882_at | Ras-like without CAAX 1 | 1.74 | 0.0165 |
| **RNPC3** | 226999_at | RNA-binding region (RNP1, RRM) containing 3 | -2.01 | 0.0473 |
| **RP11-679B1** | 235798_at | transmembrane protein 170B | -2.22 | 0.0140 |
| **RP4-691N24** | 207705_s_at | ninein-like | -1.91 | 0.0472 |
| **RP5-1022P6** | 224826_at | hypothetical protein KIAA1434 | 2.49 | 0.0410 |
| **RPL10** | 221989_at | ribosomal protein L10 | 1.52 | 0.0263 |
| **RPL11** | 200010_at | ribosomal protein L11 | 1.56 | 0.0261 |
| **RPL31** | 221593_s_at | ribosomal protein L31 | 1.52 | 0.0245 |
| **RPLP0-like** | 214167_s_at | ribosomal protein, large, P0 | 1.34 | 0.0483 |
| **RPRD2** | 226527_at | regulation of nuclear pre-mRNA domain containing 2 | -2.02 | 0.0103 |
| **RPS6** | 238156_at | ribosomal protein S6 | 2.32 | 0.0309 |
| **RSBN1** | 213694_at | round spermatid basic protein 1 | -1.95 | 0.0155 |
| **RY1** | 242607_at | small nuclear ribonucleoprotein | -1.84 | 0.0147 |
| **RYBP** | 237456_at | RING1 and YY1 binding protein | 2.32 | 0.0092 |
| **S100P** | 204351_at | S100 calcium binding protein P | 2.59 | 0.0298 |
| **SAT1** | 213988_s_at | spermidine/spermine N1-acetyltransferase 1 | 2.19 | 0.0112 |
| **SCAMP1** | 212425_at | secretory carrier membrane protein 1 | -2.10 | 0.0238 |
| **SCARA3** | 223843_at | scavenger receptor class A, member 3 | -1.90 | 0.0273 |
| **SDC2** | 212154_at | syndecan 2 | 2.82 | 0.0457 |
| **SEC14L1** | 202083_s_at | SEC14-like 1 (S. cerevisiae) | 2.24 | 0.0356 |
| **SERPINB1** | 212268_at | serpin peptidase inhibitor, clade B (ovalbumin), member 1 | 1.53 | 0.0440 |
| **SERPINB9** | 242814_at | serpin peptidase inhibitor, clade B (ovalbumin), member 9 | 2.64 | 0.0093 |
| **SERPINE1** | 202627_s_at | serpin peptidase inhibitor, clade E (nexin, plasminogen activator inhibitor type 1), member 1 | 4.32 | 0.0034 |
| **SGIP1** | 223672_at | SH3-domain GRB2-like (endophilin) interacting protein 1 | -1.63 | 0.0349 |
| **SH3GLB1** | 209091_s_at | SH3-domain GRB2-like endophilin B1 | 1.39 | 0.0433 |
| **SHROOM2** | 204967_at | shroom family member 2 | -1.91 | 0.0099 |
| **SIN3A** | 238005_s_at | SIN3 homolog A, transcription regulator (yeast) | -1.95 | 0.0144 |
| **SLA** | 203760_s_at | Src-like-adaptor | 1.79 | 0.0343 |
| **SLC11A1** | 210423_s_at | solute carrier family 11 (proton-coupled divalent metal ion transporters), member 1 | 3.10 | 0.0061 |
| **SLC16A3** | 202856_s_at | solute carrier family 16, member 3 (monocarboxylic acid transporter 4) | 2.11 | 0.0227 |
| **SLC25A37** | 242335_at | solute carrier family 25, member 37 | 2.37 | 0.0116 |
| **SLC2A12** | 244353_s_at | solute carrier family 2 (facilitated glucose transporter), member 12 | -1.91 | 0.0144 |
| **SLC2A3** | 202498_s_at | solute carrier family 2 (facilitated glucose transporter), member 3 | 3.09 | 0.0346 |
| **SLC35B4** | 225882_at | solute carrier family 35, member B4 | -1.59 | 0.0210 |
| **SLC43A3** | 213113_s_at | solute carrier family 43, member 3 | 1.83 | 0.0221 |
| **SLC6A15** | 232263_at | solute carrier family 6 (neutral amino acid transporter), member 15 | 1.87 | 0.0383 |
| **SLC7A2** | 225516_at | solute carrier family 7 (cationic amino acid transporter, y+ system), member 2 | -1.45 | 0.0433 |
| **SLCO1C1** | 220460_at | solute carrier organic anion transporter family, member 1C1 | -2.04 | 0.0148 |
| **SMAD1** | 227798_at | SMAD family member 1 | -1.59 | 0.0225 |
| **SMCHD1** | 212577_at | structural maintenance of chromosomes flexible hinge domain containing 1 | 2.06 | 0.0101 |
| **SNAPC1** | 205443_at | small nuclear RNA activating complex, polypeptide 1, 43kDa | 1.77 | 0.0195 |
| **SNRPN** | 241834_at | small nuclear ribonucleoprotein polypeptide N | -1.89 | 0.0187 |
| **SNTB2** | 226685_at | syntrophin, beta 2 (dystrophin-associated protein A1, 59kDa, basic component 2) | -1.58 | 0.0307 |
| **SNX16** | 229618_at | sorting nexin 16 | -1.50 | 0.0477 |
| **SOAT1** | 221561_at | sterol O-acyltransferase 1 | 2.47 | 0.0410 |
| **SOCS7** | 228662_at | suppressor of cytokine signaling 7 | -1.76 | 0.0148 |
| **SOD2** | 215078_at | superoxide dismutase 2, mitochondrial | 3.53 | 0.0085 |
| **SOX9** | 202936_s_at | SRY (sex determining region Y)-box 9 | -2.03 | 0.0182 |
| **SP100** | 202864_s_at | SP100 nuclear antigen | 2.02 | 0.0269 |
| **SPATA2** | 204434_at | spermatogenesis associated 2 | -1.75 | 0.0456 |
| **SPHK1** | 219257_s_at | sphingosine kinase 1 | 2.11 | 0.0040 |
| **SPSB1** | 219677_at | splA/ryanodine receptor domain and SOCS box containing 1 | 2.12 | 0.0050 |
| **SQSTM1** | 244804_at | sequestosome 1 | 2.62 | 0.0097 |
| **SRGN** | 201858_s_at | serglycin | 2.94 | 0.0161 |
| **SRPX** | 204955_at | sushi-repeat-containing protein, X-linked | 2.03 | 0.0232 |
| **STC1** | 230746_s_at | stanniocalcin 1 | 2.68 | 0.0152 |
| **STCH** | 202558_s_at | stress 70 protein chaperone, microsome-associated | 2.35 | 0.0049 |
| **STOX2** | 231969_at | storkhead box 2 | -2.10 | 0.0467 |
| **SYNCRIP** | 217834_s_at | synaptotagmin binding, cytoplasmic RNA interacting protein | 1.57 | 0.0272 |
| **SYT17** | 205613_at | synaptotagmin XVII | -1.80 | 0.0168 |
| **SYTL3** | 238423_at | synaptotagmin-like 3 | 2.46 | 0.0071 |
| **TAGAP** | 229723_at | T-cell activation RhoGTPase activating protein | 2.03 | 0.0116 |
| **TANC1** | 225308_s_at | tetratricopeptide repeat, ankyrin repeat and coiled-coil containing 1 | 1.53 | 0.0309 |
| **TANC2** | 224952_at | tetratricopeptide repeat, ankyrin repeat and coiled-coil containing 2 | -1.75 | 0.0388 |
| **TCIRG1** | 204158_s_at | T-cell, immune regulator 1, ATPase, H+ transporting, lysosomal V0 subunit A3 | 2.67 | 0.0156 |
| **TCP11L1** | 205796_at | t-complex 11 (mouse)-like 1 | 2.11 | 0.0415 |
| **TEF** | 225840_at | thyrotrophic embryonic factor | -1.84 | 0.0132 |
| **TEGT** | 200803_s_at | transmembrane BAX inhibitor motif containing 6 | 1.53 | 0.0366 |
| **TES** | 202720_at | testis derived transcript (3 LIM domains) | 2.77 | 0.0346 |
| **TFDP1** | 242939_at | transcription factor Dp-1 | 1.46 | 0.0465 |
| **TFPI2** | 209278_s_at | tissue factor pathway inhibitor 2 | 4.23 | 0.0484 |
| **TGFB2** | 228121_at | transforming growth factor, beta 2 | -1.64 | 0.0167 |
| **TGFBI** | 201506_at | transforming growth factor, beta-induced, 68kDa | 3.84 | 0.0381 |
| **TGIF1** | 203313_s_at | TGFB-induced factor homeobox 1 | 1.59 | 0.0223 |
| **THBD** | 203887_s_at | thrombomodulin | 1.88 | 0.0308 |
| **TIMP1** | 201666_at | TIMP metallopeptidase inhibitor 1 | 2.52 | 0.0375 |
| **TLR2** | 204924_at | toll-like receptor 2 | 2.27 | 0.0343 |
| **TM4SF1** | 215034_s_at | transmembrane 4 L six family member 1 | 3.79 | 0.0457 |
| **TMED5** | 202195_s_at | transmembrane emp24 protein transport domain containing 5 | 2.09 | 0.0116 |
| **TMEM178** | 229302_at | transmembrane protein 178 | -1.80 | 0.0129 |
| **TMEM38B** | 218772_x_at | transmembrane protein 38B | 1.52 | 0.0261 |
| **TMEM49** | 1569003_at | transmembrane protein 49 | 3.91 | 0.0072 |
| **TMTC3** | 226604_at | transmembrane and tetratricopeptide repeat containing 3 | -2.05 | 0.0181 |
| **TNFAIP3** | 202643_s_at | tumor necrosis factor, alpha-induced protein 3 | 3.78 | 0.0040 |
| **TNFRSF12A** | 218368_s_at | tumor necrosis factor receptor superfamily, member 12A | 2.29 | 0.0457 |
| **TNFRSF1B** | 203508_at | tumor necrosis factor receptor superfamily, member 1B | 1.83 | 0.0286 |
| **TNRC6B** | 1558142_at | trinucleotide repeat containing 6B | -1.38 | 0.0381 |
| **TPM2** | 204083_s_at | tropomyosin 2 (beta) | 1.53 | 0.0375 |
| **TPM4** | 212481_s_at | tropomyosin 4 | 2.58 | 0.0062 |
| **TRAK1** | 202080_s_at | trafficking protein, kinesin binding 1 | -1.80 | 0.0278 |
| **TREM1** | 219434_at | triggering receptor expressed on myeloid cells 1 | 2.88 | 0.0112 |
| **TRPS1** | 218502_s_at | trichorhinophalangeal syndrome I | -1.43 | 0.0383 |
| **TSHZ1** | 223282_at | teashirt zinc finger homeobox 1 | -1.76 | 0.0166 |
| **TTC8** | 227724_at | tetratricopeptide repeat domain 8 | 1.76 | 0.0201 |
| **TTLL7** | 219882_at | tubulin tyrosine ligase-like family, member 7 | 1.66 | 0.0147 |
| **TUBB6** | 209191_at | tubulin, beta 6 | 2.63 | 0.0498 |
| **TXNDC6** | 236966_at | thioredoxin domain containing 6 | -2.04 | 0.0458 |
| **TYROBP** | 204122_at | TYRO protein tyrosine kinase binding protein | 2.17 | 0.0236 |
| **UCP2** | 208998_at | uncoupling protein 2 (mitochondrial, proton carrier) | 1.61 | 0.0409 |
| **UPP1** | 203234_at | uridine phosphorylase 1 | 2.48 | 0.0393 |
| **USP3** | 221654_s_at | ubiquitin specific peptidase 3 | 1.56 | 0.0357 |
| **VAPA** | 228480_at | VAMP (vesicle-associated membrane protein)-associated protein A, 33kDa | -1.54 | 0.0326 |
| **VASP** | 202205_at | vasodilator-stimulated phosphoprotein | 1.60 | 0.0141 |
| **VCAM1** | 203868_s_at | vascular cell adhesion molecule 1 | 2.10 | 0.0098 |
| **VCL** | 200931_s_at | vinculin | 2.01 | 0.0472 |
| **VKORC1** | 217949_s_at | vitamin K epoxide reductase complex, subunit 1 | 1.59 | 0.0222 |
| **VPS13D** | 212326_at | vacuolar protein sorting 13 homolog D (S. cerevisiae) | -1.50 | 0.0358 |
| **WSB1** | 227501_at | WD repeat and SOCS box-containing 1 | 1.66 | 0.0264 |
| **XYLT1** | 213725_x_at | xylosyltransferase I | -1.74 | 0.0353 |
| **ZCCHC6** | 236243_at | zinc finger, CCHC domain containing 6 | 1.57 | 0.0311 |
| **ZFAND5** | 217741_s_at | zinc finger, AN1-type domain 5 | 1.78 | 0.0212 |
| **ZFP64** | 218968_s_at | zinc finger protein 64 homolog (mouse) | -1.49 | 0.0340 |
| **ZFYVE16** | 1555982_at | zinc finger, FYVE domain containing 16 | -1.65 | 0.0205 |
| **ZIC1** | 206373_at | Zic family member 1 (odd-paired homolog, Drosophila) | -2.04 | 0.0226 |
| **ZIC2** | 223642_at | Zic family member 2 (odd-paired homolog, Drosophila) | -1.41 | 0.0348 |
| **ZNF148** | 230821_at | zinc finger protein 148 | -1.96 | 0.0116 |
| **ZNF259** | 200054_at | zinc finger protein 259 | 1.34 | 0.0441 |
| **ZNF367** | 229551_x_at | zinc finger protein 367 | 1.57 | 0.0198 |
| **ZNF436** | 226113_at | zinc finger protein 436 | -1.86 | 0.0094 |
| **ZNF483** | 1554007_at | zinc finger protein 483 | -2.00 | 0.0139 |
| **ZNF540** | 238454_at | zinc finger protein 540 | -2.36 | 0.0029 |
| **ZNF623** | 206188_at | zinc finger protein 623 | -1.81 | 0.0101 |
| **ZNF827** | 228046_at | zinc finger protein 827 | -1.93 | 0.0180 |
| **ZXDB** | 228005_at | zinc finger, X-linked, duplicated B | -1.50 | 0.0427 |
