# Supplementary material for: Brain Perihematoma Genomic Profile Following Spontaneous Human Intracerebral Hemorrhage
Source: PLoS One. 2011 Feb 2;6(2):e16750. doi: 10.1371/journal.pone.0016750 (PMC3032742; doi:10.1371/journal.pone.0016750)
Supplement: Table S3 — Ingenuity Canonical Pathways showing a significant association [-Log(Pvalue) >1.3]. (DOC) [file pone.0016750.s004.doc]

**Table S3.**

| **Canonical Pathways** | **-Log(Pvalue)** | **Molecules** |
| --- | --- | --- |
| **TREM1 Signaling** | 6.61 | ITGB1, TLR2, IL8, TREM1, ICAM1, TYROBP, ITGA5, IL6, CCL3, ITGAX |
| **Leukocyte Extravasation Signaling** | 6.42 | ITGB1, RAC2, VCAM1, ICAM1, CDC42, MAPK8, PTK2, F11R, ITGAM, TIMP1, NCF2, CD44, GRLF1, PRKCH, VCL, CTTN, VASP |
| **Hepatic Fibrosis / Hepatic Stellate Cell Activation** | 4.68 | COL1A2, IL8, VCAM1, ICAM1, EDN1, FGF2, TIMP1, CXCL1, TGFB2, IL6, IL1R1, TNFRSF1B |
| **Caveolar-mediated Endocytosis** | 4.65 | ITGB1, B2M, ITGAM, CD55, CAV1, HLA-B, ITGA5, ITGAX, HLA-C |
| **TGFβ Signaling** | 4.27 | BMPR1B, BMP2, CREBBP, MAPK8, TGFB2, SERPINE1, SMAD1, TGIF1, INHBA |
| **Integrin Signaling** | 4.14 | ITGB1, RAC2, RALA, CDC42, ARPC5, MAPK8, ITGA5, PTK2, ITGAM, ARF4, CAV1, VCL, VASP, ITGAX |
| **Glucocorticoid Receptor Signaling** | 4.05 | IL8, VCAM1, ICAM1, CREBBP, HSPA6, MAPK8, CXCL1, IL6, HSPA5, CCL3, CD163, NFKBIA, IL1RN, TGFB2, NR3C2, SERPINE1 |
| **Hepatic Cholestasis** | 3.90 | IL8, SLCO1C1, ADCY2, NFKBIA, IL1RN, MAPK8, PRKCH, IL6, IL1R1, TNFRSF1B, IRAK2 |
| **NFkB Signaling** | 3.81 | TLR2, BMPR1B, NFKBIA, IL1RN, BCL10, BMP2, CREBBP, MAPK8, TNFAIP3, IL1R1, TNFRSF1B |
| **p38 MAPK Signaling** | 3.68 | DDIT3, IL1RN, MEF2D, TGFB2, MAP2K3, EEF2K, IL1R1, TNFRSF1B, IRAK2 |
| **IL-8 Signaling** | 3.54 | PTK2, IL8, RAC2, HMOX1, VCAM1, ICAM1, ITGAM, MAPK8, CXCL1, PRKCH, ITGAX, IRAK2 |
| **IL-10 Signaling** | 3.43 | HMOX1, NFKBIA, IL1RN, MAPK8, MAP2K3, IL6, IL1R1 |
| **IL-6 Signaling** | 3.11 | IL8, NFKBIA, IL1RN, MAPK8, MAP2K3, IL6, IL1R1, TNFRSF1B |
| **Axonal Guidance Signaling** | 3.05 | ITGB1, RAC2, PFN1, RGS3, CDC42, KALRN, BMP2, PTCH1, ARPC5, ITGA5, ADAM8, PTK2, NTRK2, SDC2, LINGO1, ABLIM2, PRKCH, VASP |
| **IL-17 Signaling** | 3.02 | IL8, TIMP1, MAPK8, CXCL1, MAP2K3, IL6, CXCL5 |
| **B Cell Receptor Signaling** | 2.99 | ETS1, PTPRC, RAC2, NFKBIA, CDC42, BCL10, EGR1, MAPK8, MAP2K3, BCL2A1 |
| **Actin Cytoskeleton Signaling** | 2.78 | PTK2, ITGB1, RAC2, ARHGEF4, FGD3, PFN1, CDC42, FGF2, ARPC5, GRLF1, ITGA5, VCL |
| **PPAR Signaling** | 2.57 | NR2F1, NFKBIA, IL1RN, CREBBP, IL1R1, TNFRSF1B, PPARGC1A |
| **PTEN Signaling** | 2.46 | PTK2, ITGB1, RAC2, BMPR1B, CDC42, ITGA5, FOXG1 |
| **Acute Phase Response Signaling** | 2.42 | HMOX1, SOD2, NFKBIA, IL1RN, MAPK8, MAP2K3, IL6, IL1R1, TNFRSF1B, SERPINE1 |
| **Fcγ Receptor-mediated Phagocytosis in Macrophages and Monocytes** | 2.40 | RAC2, HMOX1, CDC42, ARPC5, PRKCH, VASP, FGR |
| **Toll-like Receptor Signaling** | 2.40 | TLR2, NFKBIA, MAPK8, MAP2K3, IRAK2 |
| **NRF2-mediated Oxidative Stress Response** | 2.40 | HMOX1, SOD2, CREBBP, MAPK8, DNAJA4, MAP2K3, PRKCH, SQSTM1, DNAJB1, MAFF |
| **Ephrin Receptor Signaling** | 2.38 | PTK2, ITGB1, RAC2, RGS3, KALRN, CDC42, GRIN2C, SDC2, ARPC5, ITGA5 |
| **VDR/RXR Activation** | 2.18 | SERPINB1, MXD1, TGFB2, PRKCH, THBD, KLF4 |
| **PPAR****α/RXRα Activation** | 2.16 | ADCY2, NFKBIA, CREBBP, MAPK8, TGFB2, MAP2K3, IL6, IL1R1, PPARGC1A |
| **Coagulation System** | 2.15 | PLAUR, PLAU, THBD, SERPINE1 |
| **RAR Activation** | 2.06 | NR2F1, ADCY2, ALDH1A1, CREBBP, MAPK8, TGFB2, PRKCH, SMAD1, PPARGC1A |
| **CD40 Signaling** | 1.93 | ICAM1, NFKBIA, MAPK8, TNFAIP3, MAP2K3 |
| **Airway Pathology in Chronic Obstructive Pulmonary Disease** | 1.91 | IL8, CXCL1 |
| **LXR/RXR Activation** | 1.80 | MSR1, IL1RN, IL6, IL1R1, TNFRSF1B |
| **Tight Junction Signaling** | 1.73 | F11R, CDC42, VAPA, TGFB2, PRKCH, VCL, TNFRSF1B, VASP |
| **β-alanine Metabolism** | 1.68 | CNDP1, ALDH1A1, ABAT, ACADSB |
| **LPS-stimulated MAPK Signaling** | 1.67 | NFKBIA, CDC42, MAPK8, MAP2K3, PRKCH |
| **Antigen Presentation Pathway** | 1.64 | B2M, HLA-B, HLA-C |
| **CD28 Signaling in T Helper Cells** | 1.63 | PTPRC, NFKBIA, CDC42, BCL10, ARPC5, MAPK8 |
| **BMP signaling pathway** | 1.63 | BMPR1B, BMP2, CREBBP, MAPK8, SMAD1 |
| **Inositol Metabolism** | 1.56 | ERO1L, VCL |
| **Lymphotoxin β Receptor Signaling** | 1.51 | VCAM1, NFKBIA, CREBBP, CXCL1 |
| **Complement System** | 1.48 | CD59, C5AR1, CD55 |
| **Valine, Leucine and Isoleucine Degradation** | 1.44 | BCAT1, ALDH1A1, ABAT, ACADSB |
| **Death Receptor Signaling** | 1.39 | NFKBIA, MAPK8, CFLAR, TNFRSF1B |
| **Neuregulin Signaling** | 1.38 | ITGB1, DCN, ERBB4, ITGA5, PRKCH |
| **ERK/MAPK Signaling** | 1.38 | PTK2, ETS1, ITGB1, RAC2, ELF4, ITGA5, ETS2, ELK3 |
| **CCR5 Signaling in Macrophages** | 1.37 | CCL4, MAPK8, PRKCH, CCL3 |
| **Apoptosis Signaling** | 1.37 | NFKBIA, MAPK8, LMNA, TNFRSF1B, BCL2A1 |
| **Clathrin-mediated Endocytosis** | 1.34 | ITGB1, CDC42, FGF2, ARPC5, ITGA5, SH3GLB1, CTTN |
| **Activation of IRF by Cytosolic Pattern Recognition Receptors** | 1.32 | NFKBIA, CREBBP, MAPK8, IL6 |
